# The role of excitation vector fields and all-polarisation state control in cavity magnonics

**DOI:** 10.1038/s44306-024-00062-z

**Published:** 2024-12-04

**Authors:** Alban Joseph, Jayakrishnan M. P. Nair, Mawgan A. Smith, Rory Holland, Luke J. McLellan, Isabella Boventer, Tim Wolz, Dmytro A. Bozhko, Benedetta Flebus, Martin P. Weides, Rair Macêdo

**Affiliations:** 1 James Watt School of Engineering, Electronics & Nanoscale Engineering Division, University of Glasgow, Glasgow, G12 8QQ UK; 2 Department of Physics, Boston College, 140 Commonwealth Avenue, Chestnut Hill, MA 02467 USA; 3 Laboratoire Albert Fert, 1 Avenue Augustin Fresnel, 91767 Palaiseau, France; 4 Institute of Physics, Karlsruhe Institute of Technology, 76131 Karlsruhe, Germany; 5 Center for Magnetism and Magnetic Nanostructures, Department of Physics and Energy Science, University of Colorado Colorado Springs, 80918 Colorado, USA

**Keywords:** Quantum physics, Condensed-matter physics, Ferromagnetism, Spintronics

## Abstract

Recently the field of cavity magnonics, a field focused on controlling the interaction between magnons and photons confined within microwave resonators, has drawn significant attention as it offers a platform for enabling advancements in quantum- and spin-based technologies. Here, we introduce excitation vector fields, whose polarisation and profile can be easily tuned in a two-port cavity setup, thus acting as an effective experimental dial to explore the coupled dynamics of cavity magnon-polaritons. Moreover, we develop theoretical models that accurately predict and reproduce the experimental results for any polarisation state and field profile within the cavity resonator. This versatile experimental platform offers a new avenue for controlling spin-photon interactions by manipulating the polarisation of excitation fields. By introducing real-time tunable parameters that control the polarisation state, our experiment delivers a mechanism to readily control the exchange of information between hybrid systems.

## Introduction

Magnons, the quanta of spin waves in magnetic materials, have emerged as promising information carriers for developing novel classical and quantum technologies^1–3^. As a result, the field of cavity magnonics, which explores the strong coupling between magnons and photons confined within microwave cavities, has been the subject of intense research in recent years^4^. These cavity magnonic systems give rise to a quasiparticle known as the cavity-magnon polariton, and controlling the behaviour of this polariton in such systems, i.e., the hybridisation between cavity photons and magnons, offers a platform for manipulating and processing information carried by magnons. Notably, it has recently been demonstrated that magnons can couple to superconducting qubits through cavity photons and, as a consequence, cavity magnon-photon systems have garnered attention for their potential applications in quantum information processing^5–7^. Moreover, with the successful realisation and control of spin-based qubits using microwave photons, exploring and manipulating spin-photon interactions could potentially provide a powerful tool for accessing and controlling quantum states^8^. Beyond quantum computing, microwave photons are commonly used to control magnonic technologies^9^, with applications ranging from spin-based signal processing^10–12^ to magnetic sensing^13,14^ and energy-efficient computing^15,16^. Therefore, further exploiting the interaction between magnons and photons offers a promising route to the controlling, manipulating, and discerning spin systems^17^, contributing to advancements ranging from quantum technologies to magnon-based devices.

To be successful in these applications, however, it is imperative to have precise control over the coupling strength between the photons and magnons. The coupling strength dictates the rate of energy exchange, and hence controlling this rate would increase the flexibility of coherent information exchange. This has resulted in extensive efforts dedicated to engineering and controlling the coupling through various approaches^18,19^. Previous studies have explored a range of methods, including the use of various types of resonators^20–23^, modifications in materials^13,24–26^, and adjustments in sample positioning within the cavity^22,27–30^ to influence the coupling strength between magnons and photons. Additionally, researchers have developed tunable techniques that allow for transitioning between different coupling regimes without the need for physical modifications to the experimental setup^28,31–34^. Investigations into time-domain experiments, involving sequences of pulses to excite magnetic samples, have enabled dynamic control over coupling strength^35–37^, providing additional insights into the temporal aspects of magnon-photon interactions.

We demonstrate experimentally that by modifying the polarisation of cavity electromagnetic modes, one can efficiently switch from level-repulsion to coupling annihilation. In doing so, we find that different polarisation states can be readily employed to tune the coupling strength. While previous studies have explored polarisation effects on cavity magnon-polaritons including work on chiral coupling in open cavities^38,39^, in 2D cavity systems^40,41^, and by adjusting the position of a magnetic sample inside a cavity where degenerate modes of different polarisations exist^42,43^, our work presents a flexible approach to on-demand control of polarisation states within a 3D cavity resonator. Our study offers an automated method for adjusting polarisation states without physical repositioning of cavity components and a comprehensive exploration of how the polarisation state of the excitation vector fields within the resonator can modify the coupling of the hybrid modes. We show how a square cavity resonator—excited by two ports—can be used to generate multiple polarisation states, namely circularly, linearly and elliptically polarised modes, and these can dramatically modify the overall behaviour of the cavity-magnon hybridisation. For instance, we find that by using circularly polarised excitation fields, we can transition from complete decoupling to an enhancement in the coupling strength by a factor of $\sqrt{2}$ in comparison to excitation by a linearly polarised field. We provide two theoretical frameworks—namely, electromagnetic perturbation theory and a quantised input-output theory—that both accurately predict the behaviour of polarisation-dependent cavity magnon-polaritons. In what follows, we start by discussing electromagnetic perturbation theory as a way to build intuition for the problem. This theory also allows us to draw direct parallels with well-known concepts such as that of intrinsic handedness of magnetisation precession as well as to calculate the coupling strength without any need for experimental fitting parameters. While intuitive, pertubation theory on its own can only be used to estimate the coupling strength, therefore, by employing a second quantisation model we are also able to obtain reflection (S-parameter) spectra whilst describing the system in the familiar language of magnon-polaritons. Nonetheless, both models are equivalent and as we will see, describe our experimental data extremely accurately. Moreover, through the polarisation state we are able to engineer an external applied magnetic field non-reciprocity mechanism into cavity-magnon hybridisation, adding yet another tunability mechanism to our system. Finally, we expect that by being able to fully control and understand the behaviour of these polarisation-dependent systems, we hold considerable promise for realising tunable magnonic devices and enabling advancements in quantum information processing and spin-based technologies.

## Results

The premise of our study is quite simple: spin precession has an inherent chirality, as such, to effectively excite magnetisation a driving excitation polarised with compatible chirality is needed. This fundamental principle forms the basis of our investigation as it should extend to the coupling between magnons and photons inside a microwave cavity giving rise to polarisation-dependent cavity magnon-polaritons.

### Right and left circularly polarised cavity magnon-polaritons

We start by building context and positioning our findings within classic literature that combines spin precession and electromagnetic theory. The magnetic response of a material to an applied magnetic field can be characterised classically by the Landau-Lifshitz (LL) equation^44^. If we take the case where an external static magnetic field *H*_0_ is applied along the *z* direction and a time-varying AC field oscillating the *x*-*y* plane, represented by **h***e*^*iωt*^, then the effective field can be written as ${{\bf{H}}}_{{\bf{eff}}}=\hat{{\bf{z}}}{H}_{0}+{\bf{h}}{e}^{i\omega t}$ (where the observed magnetic field corresponds to the real part of **H**_**eff**_). Upon solving the LL equation for a ferromagnetic sphere, which can be approximated to a macrospin, the following relation between the oscillating magnetisation **m** and the oscillating magnetic field is derived:

1 $$\left[\begin{array}{c}{m}_{x}\\ {m}_{y}\end{array}\right]=\underbrace{\left[\begin{array}{cc}{\chi }_{a}&i{\chi }_{b}\\ -i{\chi }_{b}&{\chi }_{a}\end{array}\right]}\limits_{{\overleftrightarrow{\chi }}_{m}(\omega )}\left[\begin{array}{c}{h}_{x}\\ {h}_{y}\end{array}\right].$$

Here, ${\overleftrightarrow{\chi }}_{m}(\omega )$ is the high-frequency magnetic susceptibility which is a non-symmetric second rank tensor and its components have the form ${\chi }_{a}={\omega }_{0}{\omega }_{m}/({\omega }_{0}^{2}-{\omega }^{2})$ and ${\chi }_{b}=\omega {\omega }_{m}/({\omega }_{0}^{2}-{\omega }^{2})$; where *ω*_0_ = *μ*_0_*γH*_0_ is the natural precession frequency of a magnetic dipole in a constant magnetic field, and *ω*_*m*_ = *μ*_0_*γM*_*s*_.

**Fig. 1:**
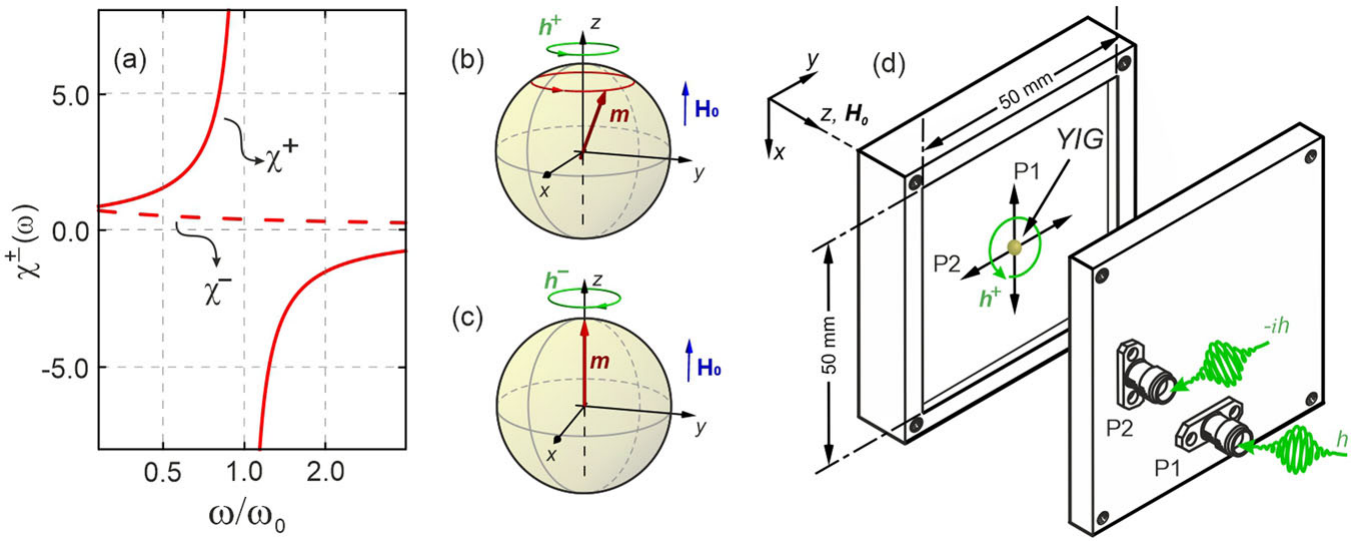
Compatibility of the handedness of the magnetic driving field with the intrinsic handedness of the magnetisation precession. **a** Effect of right **h**^+^ and left **h**^−^ circular polarised excitation on the susceptibility *χ*^±^ = *ω*_*m*_/(*ω*_0_ ∓ *ω*). The lines for *χ*^±^ were calculated using the magnetic parameters for YIG: *μ*_0_*M*_*s*_ = 0.1758 T, *γ* = 28 GHz/T, and *H*_0_ = 230 mT (corresponding to *ω*_0_ = 6.44 GHz). The sphere diagrams in (**b**) and (**c**) illustrate the effect of the polarisation of the driving field, **h**, on the magnetisation, **m**. **b** Magnetisation excited by a right circularly polarised field inducing a strong response (large precession cone) and (**c**) vanishingly-small precession seen for a left circularly polarised excitation. **d** Diagram of the cavity used to generate excitation fields with different polarisations. By controlling the amplitude and phase of the input signals to port 1 (P1) and port 2 (P2), excitation fields with any polarisation can be generated at the sample position (refer to Supplementary Material E for more detail).

Now, let the driving field be right- or left-circularly polarised, so that it can be written as

$${{\bf{h}}}^{+}(t)=(\hat{{\bf{x}}}h-\hat{{\bf{y}}}ih){e}^{i\omega t}$$

or

$${{\bf{h}}}^{-}(t)=(\hat{{\bf{x}}}h+\hat{{\bf{y}}}ih){e}^{i\omega t},$$

respectively. Eq. (1) can then be rewritten with **m** and **h** as circularly polarised quantities, and it takes the form:

2 $${{\bf{m}}}^{\pm }=\frac{{\omega }_{m}}{{\omega }_{0}\,\mp\, \omega }{{\bf{h}}}^{\pm },$$

where **m**^±^ = *m*_*x*_ ± *m*_*y*_ and **h**^±^ = *h*_*x*_ ∓ *ih*_*y*_. One of the well-known consequences of this is that for right-circularly polarised driving field **h**^+^, the susceptibility *χ*^+^ = *ω*_*m*_/(*ω*_0_ − *ω*) has a singularity (resonance) at *ω* = *ω*_0_ [shown as a solid line in Fig. 1a]. In this case, a large response takes place as the oscillating magnetisation and the excitation vector fields are in phase. This is known as the Larmor precession (or Larmor condition) and is sketched in Fig. 1b as a large-angle magnetisation precession cone. On the other hand, the left-circularly polarised field **h**^−^, has a sense of rotation opposite to the magnetisation’s natural precession, and thus the Larmor condition cannot be met. For this case, the susceptibility *χ*^−^ = *ω*_*m*_/(*ω*_0_ + *ω*) has no singularity and a much smaller magnitude than *χ*^+^ [shown as the dashed line in Fig. 1a]. This behaviour is also depicted in Fig. 1c, and is known as “anti-Larmor” precession^45,46^. This behaviour is a direct consequence of the handedness of spin precession (i.e., the clockwise or anti-clockwise direction of precession is dictated by the direction of **H**_**0**_). It should be noted that reversing the direction of **H**_**0**_ would cause the signs in the denominator of Eq. (2) to flip, changing from ∓ to ±. Thus, **h**^+^ would then correspond to a left-circularly polarised excitation with respect to **H**_**0**_ [Eq. (2) would have no singularity], while **h**^−^ would correspond to right-circularly polarised with respect to **H**_**0**_ [Eq. (2) would have a singularity at *ω* = *ω*_0_].

To ascertain how the behaviour detailed above affects cavity magnon-polariton systems, we have designed a three-dimensional microwave resonator [shown in Fig. 1d] where the polarisation state of the microwave, cavity-bound photons can be controlled using a two-port set up. The position of these ports is designed so that—in combination with direct control of the amplitude and phase of the input at each port—the superposition of the excitation vector fields allow for a tunable, all-polarisation state system (at the sample position). This allows us to experimentally probe the coupling between magnons and cavity photons by placing a Yttrium Iron Garnet (YIG) sphere in the centre of the cavity where the excitation vector fields can be controlled to be left or right circularly polarised by controlling the phase of one of the inputs.

**Fig. 2:**
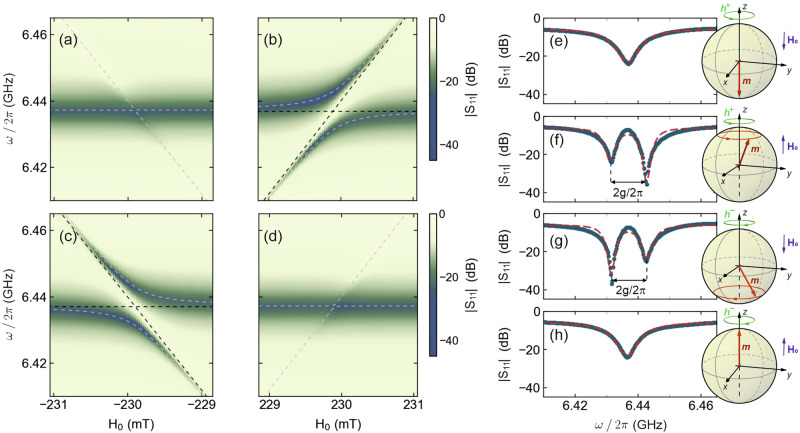
Cavity magnon-polariton response to circularly polarised excitation fields and bias fields applied along the $\pm \hat{{\bf{z}}}$ axis. Experimental spectra and perturbation theory (dashed grey lines) of the quasi-Rabi splitting close to *ω*_0_ = *ω*_*c*_ for (**a**) ${{\bf{H}}}_{{\bf{eff}}}=-\hat{{\bf{z}}}{H}_{0}+{{\bf{h}}}^{+}$; (**b**) ${{\bf{H}}}_{{\bf{eff}}}=\hat{{\bf{z}}}{H}_{0}+{{\bf{h}}}^{+}$; (**c**) ${{\bf{H}}}_{{\bf{eff}}}=-\hat{{\bf{z}}}{H}_{0}+{{\bf{h}}}^{-}$ and (**d**) ${{\bf{H}}}_{{\bf{eff}}}=\hat{{\bf{z}}}{H}_{0}+{{\bf{h}}}^{-}$. The dashed black lines show the cavity mode and Kittel mode. **e**–**h** show the corresponding ∣*S*_11_∣ parameter measured when *ω*_0_ = *ω*_*c*_. The sphere diagrams illustrate the direction of the magnetic bias field, the excitation field and the subsequent spin precession in the YIG.

The experimental spectra displayed in Fig. 2 were obtained by controlling both the excitation field polarisation state and the direction of the bias field, **H**_**0**_. We show examples for left, **h**^−^, and right, **h**^+^, circularly polarised excitation vector fields for bias fields of $\pm \hat{{\bf{z}}}{H}_{0}$. Comparing Fig. 2a, b, we can see that by simply reversing the direction of the **H**_**0**_, it is possible to go from level crossing (a) to level repulsion (b). In the first case, the modes cross each other and no hybridisation takes place while in the second hybridisation takes place between both systems and an ‘anti-crossing’ of the modes is observed. In Fig. 2c, d the opposite behaviour is observed; namely, − **H**_**0**_ shows avoided crossing while + **H**_**0**_ shows level crossing. This is achieved by changing the sense of rotation of the circularly polarised excitation vector field with respect to the direction of **H**_**0**_.

These results can be reproduced using well-established perturbation theory (See Supplemental Material A), as first reported in the work by Artman and Tannenwald^47^. By measuring the magnetic susceptibility of ferrite samples, they demonstrated how the chirality of spin precession can be probed by circularly polarised driving fields. This theory uses Maxwell’s equations to obtain the following relation to estimate the shift in the cavity resonance frequency *ω*_*c*_ due to a small perturbation^27^:

3 $$\frac{\omega -{\omega }_{c}}{{\omega }_{c}}=-\frac{{\int}_{\delta v}{\mu }_{0}[{\overleftrightarrow{\chi }}_{m}(\omega )\cdot {{\bf{h}}}_{{\bf{c}}}]\cdot {{\bf{h}}}_{{\bf{c}}}^{* }{\rm{d}}v}{2{\int}_{v}{\mu }_{0}| {{\bf{h}}}_{{\bf{c}}}{| }^{2}{\rm{d}}v},$$

where **h**_**c**_ denotes the oscillating magnetic field of the cavity, *δv* is the sample volume and *v* is the volume of the empty cavity. Up to now, perturbation theory has been limited to describing the coupling between magnons and microwave photons that are linearly polarised. However, if we introduce circularly polarised excitation vector field by making **h**_**c**_ = **h**^±^ at the sample position we can rewrite Eq. (3) as

4 $$\frac{\omega -{\omega }_{c}}{{\omega }_{c}}=-\frac{{\omega }_{m}}{{\omega }_{0}\,\mp\, \omega }\frac{{\int}_{\delta v}| {{\bf{h}}}^{\pm }{| }^{2}{\rm{d}}v}{2{\int}_{v}| {{\bf{h}}}_{{\bf{c}}}{| }^{2}{\rm{d}}v}$$

where ‘ − ’ in the denominator is for **h**^+^ and ‘ + ’ is for **h**^−^ (both defined with respect to ${{\bf{H}}}_{{\bf{0}}}=+\hat{{\bf{z}}}{H}_{0}$). Note that we cannot replace **h**_**c**_ by **h**^±^ in the denominator as the integral is evaluated across the whole volume of the cavity and at some positions, unlike at the centre, **h**_**c**_ may not add up to a circularly polarised quantity. These imply that a circularly polarised microwave magnetic field with the same chirality as the precession motion has a singularity at *ω* = *ω*_0_; here corresponding to level repulsion. On the other hand, the response of the medium to an excitation with opposite chirality to the magnetisation is non-resonant [Eq. (4) and has no singularity if we take the ‘ + ’ sign], which corresponds to coupling annihilation as the solutions of Eq. (4) yields simply the decoupled *ω*_*c*_ and *ω*_0_ frequencies. This is not only in agreement with the experimental results but also directly reflects the behaviour discussed in Fig. 1.

By solving Eq. (4) for *ω* ≊ *ω*_*c*_, *ω*_0_, we obtain the eigenfrequencies of the cavity-magnon hybrid system. By using the magnetic parameters for YIG [same as used in Fig. 1a] we obtain the eigenfrequencies for the two modes (branches), which are in excellent agreement with the experimental contour data. In Fig. 2e–h, we show cross-sectional ∣*S*_11_∣ spectra taken from the data in the maps shown in (a)–(d). These are taken at a *H*_0_ value corresponding to *ω*_*c*_ = *ω*_0_ since the width of the splitting between the eigenfrequencies at this point is related to the macroscopic coupling strength, *g* (2*g* = *Δω*)^48^. From the plots, we can obtain an experimental value for *Δω*, and thus the coupling strength, *g*. Notably, in the case of Fig. 2e, h, the cavity and magnon modes coalesce, i.e., indistinguishable eigenmodes. However, for Fig. 2f, g, we measure *Δω*/2*π* of 11 MHz experimentally. Conspicuously, we have a large *Δω* for level repulsion, signifying strong coupling, whereas *Δω* = *g* = 0 for level crossing, indicating coupling annihilation. We note that the above analysis has neglected damping of both cavity and magnon modes. While these can be phenomenologically included in Eq. (4)^27^, since both YIG and the cavity have extremely small dissipation rates, their effect on perturbation theory eigenfrequencies can be neglected.

Furthermore, it is important to note that field non-reciprocity can be induced in our system. For instance, in Fig. 2e, f, we observe the interaction of a right-circularly polarised excitation, **h**^+^, with spin precession when a static magnetic field, **H**_**0**_, is applied along $\pm \hat{{\bf{z}}}$. In Fig. 2f, the resonance condition is met as both the wave, **h**^+^, and the magnetisation precession have the same chirality and level splitting is observed (two distinct dips) at *ω*_0_ = *ω*_*c*_. In Fig. 2e on the other hand, the condition is not met as torque now dictates that precession motion has opposite chirality to **h**^+^ and thus only one dip is observed (corresponding to *ω*_*c*_ as *ω*_0_ is not excited). Effectively, by not changing the chirality of the excitation vector field but reversing the sign of **H**_**0**_, it is possible to switch from strong spin precession to almost no precession at all and consequently from strong coupling to coupling annihilation. The opposite behaviour then takes place for **h**^−^; namely strong coupling for when **H**_**0**_ is applied along $-\hat{{\bf{z}}}$ [Fig. 2g] and coupling annihilation when **H**_**0**_ is applied along $+\hat{{\bf{z}}}$ [Fig. 2h].

### Tunable magnon-polariton coupling

Thus far, our analysis has primarily focused on the special case of circularly polarised excitation vector fields. However, by implementing the experimental set-up shown in Fig. 1d, we establish a robust system that enables precise control of the polarisation of the driving excitation vector field. This allows us to engineer a continuous change in polarisation states (from linearly to circularly and elliptically polarised excitations) by controlling both the relative amplitude and phases between the inputs in both cavity ports so that the oscillating driving field is:

5 $${{\bf{h}}}_{c}=(\hat{{\bf{x}}}+\hat{{\bf{y}}}\delta {e}^{i\varphi })h{e}^{i\omega t}$$

where *δ* is the amplitude ratio and *φ* is the relative phase between the ports (and hence *x* and *y* components of **h**_**c**_ at the sample position) and with observable field corresponding to Re(**h**_**c**_). The amplitude ratio *δ* and relative phase *φ* are defined as ∣*h*_*cy*_∣/∣*h*_*cx*_∣ and $\arg ({h}_{cy})-\arg ({h}_{cx})$ respectively. For context, in this general case, when the amplitude ratio *δ* = 1 (meaning both components of the excitation field have the same amplitudes) and a phase difference of *φ* = −90°, it corresponds to a right-circular polarised excitation (**h**^+^), while a phase difference of *φ* = + 90° corresponds to left-circular polarised excitation, (**h**^−^). Because we have precise control of the values of *δ* and *φ*, other intermediate polarisation states of the excitation vector fields can also be introduced, such as elliptically or linearly polarised states.

This can be readily implemented into Eq. (3) to obtain a general expression similar to Eq. (4) but it instead takes into account any polarisation state (See Supplemental Material A). However, perturbation theory, by itself, is limited to calculating eigenfrequencies—which can in turn be used to estimate the coupling strength. Therefore, to quantify and analyse this general case in terms of magnon-polariton modes, we proceed to quantise the cavity fields. Unlike electromagnetic perturbation theory, this method relies on more experimental fitting parameters. However, its versatility and completeness allows us not only to calculate the eigenfrequencies of the system but also to directly calculate the reflection (S-parameter) where both magnon damping and cavity losses play a vital role and for the remainder are both included properly. The Hamiltonian of the microwave cavity interacting with the magnetic sphere can be written as

6 $$H={\int}_{\!v}\left({\mu }_{0}{{{\bf{h}}}_{{\bf{c}}}}^{2}\right){d}^{3}r-{\int}_{\!\delta v}\left({\mu }_{0}{M}_{s}{H}_{0}\right){d}^{3}r-{\int}_{\!\delta v}{\mu }_{0}\left({\bf{m}}\cdot {{\bf{h}}}_{{\bf{c}}}\right){d}^{3}r.$$

The first term in Eq. (6) denotes the energy of the cavity of volume *v* with associated magnetic field **h**_**c**_ while the second term represents the Zeeman energy. As discussed previously, the static magnetic field *H*_0_ is taken to be along $\hat{z}$. The final term in Eq. (6) constitutes the cavity-magnon interaction. Here, **m** = *γ*ℏ**S**/*δv* is the magnetization of a YIG sphere of volume *δv* with $S\equiv | {\bf{S}}| =\frac{5}{2}\rho \delta v$ being the coarse-grained, classical spin obtained upon averaging the spin density *ρ* over the complex unit cell of a material such as YIG (note that the magnetic moment of a YIG arises from the Fe^3+^ ions, wherein, two of five iron spins and the remaining three in a unit lattice orient the opposite direction with a net spin *s* = 5/2^49,50^ with a spin density that goes as *ρ* ≈ 4.22 × 10^27^). The cavity magnetic field **h**_**c**_ can then be associated with the quantised vector potential of the electromagnetic field in Coulomb gauge^51–53^, which allows to rewrite

7 $${{\bf{h}}}_{{\bf{c}}}=i\sqrt{\frac{2\pi \hslash \omega_c }{v}}\hat{\epsilon }f(\overrightarrow{r}){e}^{-i\omega t}a+\,\text{h.c.}\,,$$

where $\hat{\epsilon }={\epsilon }_{x}\hat{x}+{\epsilon }_{y}\hat{y}$ with ∣*ϵ*_*x*_∣^2^ + ∣*ϵ*_*y*_∣^2^ = 1, *ω*_*c*_ denotes the cavity resonance frequency, $f(\overrightarrow{r})$ describes the spatial mode of the cavity field and *a* (*a*^†^) characterises the annihilation (creation) operators of the cavity field. To describe our experimental setup, in which, the driving magnetic field at the sample position is prepared in a superposition of $\hat{x}$ and $\hat{y}$ with an overall phase shift *φ* and amplitude *δ* between *h*_*cx*_ and *h*_*cy*_, we define the field polarisation as

8 $$\hat{\epsilon }=\frac{\delta {e}^{i\varphi }\hat{x}+\hat{y}}{\sqrt{1+{\delta }^{2}}}.$$

We note that this is equivalent to describing the cavity as driven by two orthogonally polarised modes, ${a}_{\vec{k}x}$ and ${a}_{\vec{k}y}$, as shown in Supplementary Note B. While this description may seem more akin to our experimental set up, the superposition of these modes still produces a single effective cavity mode *a*. Therefore, we proceed our discussion in terms of *a* only. Employing the Holstein-Primakoff transformation in the dilute magnon limit (since the temperature is far below YIG critical temperature)^54^, we can bosonize the spin system by rewriting

9 $${S}_{z}=S-{b}^{\dagger }b,\quad {S}_{+}={S}_{x}+i{S}_{y}=\sqrt{2S-{b}^{\dagger }b}b\approx \sqrt{2S}b,$$

where *b*(*b*^†^), with [*b*, *b*^†^] = 1, denotes the annihilation (creation) operator of the Kittel mode, i.e., the uniformly precessing magnon mode. Under the rotating wave approximation, using Eqs. (7)–(9)), we can rewrite Eq. (6) as

10 $$H=\hslash \omega {a}^{\dagger }a+\hslash {\omega }_{0}{b}^{\dagger }b+\hslash [\tilde{g}b{a}^{\dagger }+{\tilde{g}}^{* }a{b}^{\dagger }].$$

For simplicity, $\tilde{g}$ has been introduced to represent the coefficients multiplying the terms *ba*^†^ and *ab*^†^, obtained after applying the rotating wave approximation and the substitutions from Eqs. (7)–(9)) to Eq. (6). Thus, $\tilde{g}$ is the effective magnon-photon coupling given by

11 $$\tilde{g}=\frac{g}{\sqrt{1+{\delta }^{2}}}(1-i\delta {e}^{i\varphi })$$

with $g=\gamma \eta \sqrt{5N\pi \hslash \omega /(2v)},N=\rho \delta v$ and the spatial overlap integral $\eta =\frac{1}{\delta v}{\int}_{\delta v}f(\overrightarrow{r}){d}^{3}r$.

Finally, the Hamiltonian of the system, incorporating the interaction of the cavity with the probe field is given by

12 $$H=\hslash \omega {a}^{\dagger }a+\hslash {\omega }_{0}{b}^{\dagger }b+\hslash \widetilde{g}{a}^{\dagger }b+\hslash {\widetilde{g}}^{* }a{b}^{\dagger }+i\hslash {\epsilon }_{c}({a}^{\dagger }{e}^{-i{\omega }_{c}t}-a{e}^{i{\omega }_{c}t}),$$

where the last term on the right-hand side of Eq. (12) describes the electromagnetic probe field at frequency *ω*_*c*_ and power *D*_*c*_, where ${\epsilon }_{c}=\sqrt{2\kappa {D}_{c}/(\hslash {\omega }_{c})}$ parameterises the strength of the probe field and *κ* depicts the effective decay of the mode *a* into the electromagnetic vacuum.

Here we use the input-output relation (Refer to Supplemental Material B)

13 $${a}_{in}+{a}_{out}=\sqrt{2\kappa }a.$$

where *a*_*in*_ and *a*_*out*_ can be interpreted as the incoming excitation into the cavity and outgoing signal, respectively. Therefore, ${a}_{in}={\varepsilon }_{c}/\sqrt{2\kappa }$.

The dynamics of the system in the frame rotating at frequency *ω*_*c*_ is governed by the following Heisenberg equations (written in a matrix form):

14 $$\left[\begin{array}{c}\dot{a}\\ \dot{b}\end{array}\right]=-i\left[\begin{array}{cc}{\omega }_{c}-\omega -i\kappa &\widetilde{g}\\ {\widetilde{g}}^{* }&{\omega }_{0}-\omega -i\eta \end{array}\right]\left[\begin{array}{c}a\\ b\end{array}\right]+\left[\begin{array}{c}{\varepsilon }_{c}\\ 0\end{array}\right].$$

where *η* is the decay rate of the Kittel mode of the YIG sphere.

In the long-time limit, we can solve Eq. (14) to obtain *a*. Substituting this into Eq. (13), we find the reflection coefficient to be

15 $$| {S}_{11}(\omega )| =\left| \frac{{a}_{out}}{{a}_{in}}\right| =\left| \frac{i\kappa ({\omega }_{0}-\omega -i\eta )}{(\omega -{\omega }_{c}+i\kappa )({\omega }_{0}-\omega -i\eta )+| \tilde{g}{| }^{2}}-1\right| .$$

It is worth mentioning that the *φ* dependence of *S*_11_ from the $| \tilde{g}{| }^{2}$ in the denominator is identical to the *φ* dependence in Eq. (S3), ensuing from the full perturbation theory discussed in the supplementary material, further corroborating the equivalence of both methods.

**Fig. 3:**
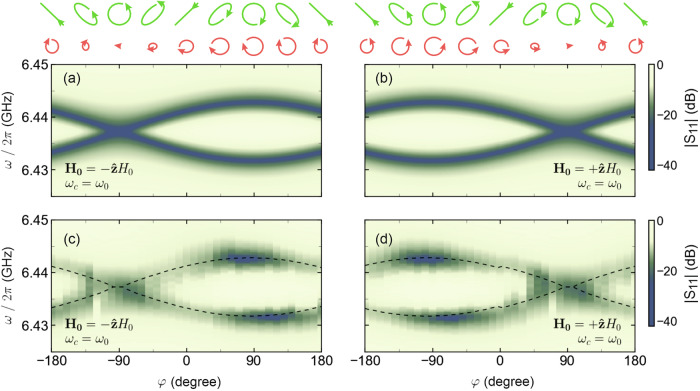
Tunable hybridisation of cavity magnon-polaritons. The hybridisation behaviour at *ω*_0_ = *ω*_*c*_ for a bias field of $+\hat{{\bf{z}}}{H}_{0}$ in (**a**) and $-\hat{{\bf{z}}}{H}_{0}$ in (**b**) as *φ* is changed and *δ* = 1. The values were calculated from Eq. (15) using the experimentally measured parameters *κ*/2*π* = 13.25 MHz, *η*/2*π* = 0.7 MHz (see Supplemental Material D) and *g*/2*π* = 3.9 MHz. The experimental amplitudes of the ∣*S*_11_∣ parameter for various values of *φ* when *ω*_0_ = *ω*_*c*_ and *δ* = 1 is shown for a bias field of $+\hat{{\bf{z}}}{H}_{0}$ in (**c**) and $-\hat{{\bf{z}}}{H}_{0}$ in (**d**). The dashed black lines are the eigenfrequencies extracted from (**a**) and (**b**). The diagram above the figure illustrates the excitation field polarisation (depicted as the green arrows) and the subsequent precession cone (depicted as the red arrows) as *φ* is changed, for *δ* = 1, calculated from the LL equation.

From Eq. (15), and from the expression for $\tilde{g}$, one can expect that by manipulating the polarisation of the cavity excitation vector fields, the cavity-magnon interaction can be controlled. To demonstrate this, in Fig. 3, we show a map of ∣*S*_11_(*ω*)∣, comparing spectra at *ω*_0_ = *ω*_*c*_ and *δ* = 1 for a 360° phase scan, illustrating, how to tune the Rabi splitting by simply varying *φ*, even when the amplitude of both *h*_*cx*_ and *h*_*cy*_ are identical. In Fig. 3a, b we show calculated values of transmission coefficient using Eq. (15) which are directly compared with experimental data given in Fig. 3c, d. The theory shows remarkable agreement with the experimental contours and from both cases, it is evident that the Rabi splitting increases as we move from *φ* = 0 to −90° and decreases towards the annihilation point at *φ* = + 90° for $+\hat{{\bf{z}}}{H}_{0}$ [Fig. 3b, d]. These points correspond to **h**^+^ and **h**^−^, respectively and are defined with respect to the direction of *H*_0_—the cases discussed in Fig. 1. The diagram above the figure depicts the polarisation of the excitation state for various *φ* (green) and the subsequent magnetic precession in the sample (red). For a bias field of ${{\bf{H}}}_{{\bf{0}}}=+\hat{{\bf{z}}}{H}_{0}$, we observe a significant decrease in the precession cone at +90°, consistent with previous findings indicating no coupling due to the opposite chirality of the excitation field relative to the magnetisation precession handedness. As the phase is swept to −90°, the chirality of the magnetic excitation field progressively matches the intrinsic handedness of the magnetisation precession. At *φ* = −90°, both the right-circularly polarised excitation field and magnetisation have the same chirality, enabling maximal driving of the precession dynamics. In addition to the cases of circularly polarised excitation, other special cases include *φ* = −180°, 0°, 180° which result in a linearly polarised **h**_**c**_. The result for these cases is somewhat similar to right circular polarisation, however, the precession cone is smaller for linear polarisation due to the weaker coupling between the excitation field and magnetisation. For linearly polarised excitation, we obtained a *Δω*/2*π* = 7.8 MHz (which is a factor of $1/\sqrt{2}$ smaller when compared with the *Δω* for **h**^+^). Moreover, any other case then represents elliptical polarisation; where the behaviour is analogous to that of circularly polarised states (in that the handedness is important) albeit the coupling is not as strong.

Interestingly, switching the magnetic bias field effectively [$-\hat{{\bf{z}}}{H}_{0}$, shown in Fig. 3a, c] induces a 180° phase shift in the wave’s rotation, as anticipated. Namely, magnetisation is now excited at *φ* = +90° which corresponds to a right circularly polarised excitation with respect to the direction of *H*_0_. Similarly, the response of the magnetisation greatly decreases, and vanishes, as *φ* approaches −90°. This is because the excitation field is left circularly polarised in relation to the magnetisation and no component of **h**_**c**_ is able to excite spin precession.

So far, we have been examining the impact of elliptically polarised excitation fields with an amplitude ratio of *δ* = 1. However, our versatile experimental set-up [see Fig. 5] allows us to explore how the system responds to various excitation fields with all possible polarisation states. In turn, we can fully understand and control the hybridisation behaviour with excitation fields of any polarisation state. To this end, we investigate the effect of varying *δ* and *φ* to summarise the behaviour of *Δω* for all different excitation conditions. It is important to note that in this experiment, we vary the amplitude ratio within the range 0 ≤ *δ* ≤ 1. This is because *δ* ranging from 1 to infinity corresponds to driving fields with the same polarisation state as *δ* spanning from 1 to 0. Thus, in this parameter space, we expect the same hybridisation behaviour (more details are provided in Supplemental Material F).

**Fig. 4:**
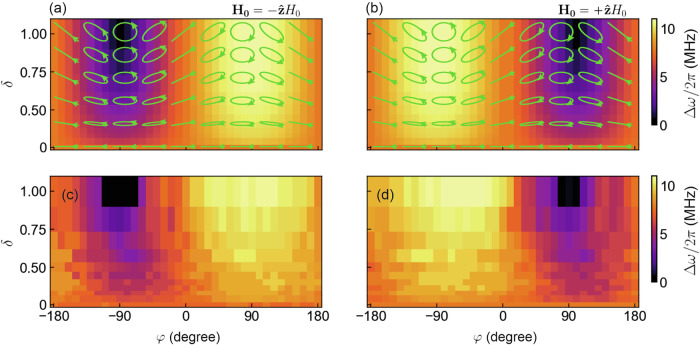
Summary of the dependence of cavity magnon-polariton coupling behaviour on excitation field polarisation. A heat map summarising the width of the quasi-Rabi splitting, *Δω*/2*π*, as a function of *δ* and *φ* calculated from Eq. (15) for a bias field of $+\hat{{\bf{z}}}{H}_{0}$ in (**a**) and $-\hat{{\bf{z}}}{H}_{0}$ in (**b**). Shown in green, are the polarisation states corresponding to various *δ* and *φ*. The experimentally measured quasi-Rabi splitting, *Δω*/2*π*, for various *δ* and *φ* for the same bias fields are shown in (**c**) and (**d**) respectively.

From Fig. 4, we observe the potential to transition between different coupling regimes solely by adjusting the relative phase and amplitude between the two field components – where any arbitrary value of *δ* and *φ* will correspond to an elliptically polarised excitation. The arrows in Fig. 4a, b illustrate the polarisation of the excitation field for various *δ* and *φ* and the heat maps present the theoretical values of *Δω*/2*π* obtained from Eq. (11) for the corresponding excitation fields, providing a comprehensive mapping of the coupling behaviour across the parameter space of *δ* and *φ*. The experimental results investigating the amplitude ratio and phase effects are presented in Fig. 4c, d. As the amplitude ratio *δ* approaches 0, the excitation field becomes increasingly linearly polarised, as shown in the arrow diagrams. In this regime, as *δ* ≈ 0, the field is effectively linear with only *x* being the dominant component, making the phase between the *x*- and *y*-components, *φ*, irrelevant. At *δ* = 0, we measured a *Δω*/2*π* of 7.8 MHz, consistent across all values of *φ*. This result aligns with the analytical prediction from Eq. (11), which indicates that as *δ* approaches 0, the effective magnon-photon coupling strength simplifies to the value expected for linearly polarised excitation ($\tilde{g}\approx g$), with no dependence on *φ*. The predicted *Δω*/2*π* of 7.8 MHz from Eq. (11) thus demonstrates excellent agreement with our experimental findings for *δ*=0. While our experimental setup is limited to *δ* ≤ 1, we expect a similar behaviour for *δ* > 1; namely, as *δ* increases beyond 1, the polarisation of the excitation field becomes increasingly linear, dominated by the *y*-component, rendering the phase *φ* between the two components irrelevant (as the *x*-component becomes negligible). Consequently, in the limit where *δ* → *∞*, we expect the effective coupling strength $\tilde{g}\approx g$ for all *φ*, mirroring the behaviour observed at *δ* ≈ 0, as predicted by Eq. (11).

We can also see that, for the special case when the excitation field is right circularly polarised with respect to **H**_**0**_, the coupling reaches its maximum value. Conversely, when the excitation field is left circularly polarised relative to **H**_**0**_, we clearly observe no coupling. These findings are in excellent agreement with the results discussed in Figs. 2 and 3. Furthermore, by setting *φ* to an arbitrary value, such as ± 90°, we demonstrate that the coupling strength can be modulated simply by varying *δ*. This highlights how *δ* serves as an additional control parameter for tuning the magnon-photon interaction.

### Discussion

Here, we have shown how the excitation vector field can dramatically alter magnon-cavity hybridised states, i.e., cavity magnon-polaritons. By all-polarisation control over the excitation vector field, it is possible to not only enhance the coupling but also annihilate it depending on the sense of rotation of the excitation.

The understanding presented here is particularly relevant for technological applications based on cavity magnonics, where controlling the coupling between magnons and photons is crucial. For instance, they are expected to aid bidirectional conversion between radio-frequency waves and light^55,56^. Moreover, as cavity magnon-polaritons can also couple with qubits^6^, controlling the interaction between magnons and photons enables the ability to coherently exchange of information between qubits and cavity magnon-polaritons, thereby providing a valuable path for quantum information processing^5,7^. In both cases, engineering as well as understanding the coupling are crucial steps to optimise the conversion and (or) information exchange. In our work, we engineered a two-port cavity system where the nature of coupling can be tuned at will. Through the theoretical framework presented here, we also are able to predict as well as gain further insight into the nature of the coupling between microwave cavities and magnons in a rigorous manner for any excitation field. Recent work has also used two port cavities such as we do here beyond controlling strong coupling and to obtain another regime of coupling altogether: level attraction^57^. In our case, we have obtained spectra resembling level attraction (See Supplementary Note F) due to interference between transmission and reflection spectra, which is a limitation of how this set up can generate polarisation. However, as shown in Ref. ^57^ this versatile experiment can be used well beyond polarisation investigation. Similar conclusions regarding level attraction attributed to the interference, albeit between travelling and standing modes, have also been reported by Yao et al.^58^.

It is also important to point out here that field non-reciprocity can be induced in our system. A qualitatively similar behaviour to the non-reciprocity for circularly polarised waves has recently drawn the attention of the optics and photonics community in the context of cavity quantum electrodynamics. In this case, however, the non-reciprocity is obtained through time-reversal symmetry break in Fabry-Pérot cavities^59^. While in our case we do not have time-reversal symmetry breaking—as reversal of time would change both the direction of **H**_**0**_ and sense of rotation of **h**_**c**_—the field reversal behaviour is still of particular interest for non-reciprocal devices as it can enable tunable devices that can readily switch between regimes of hybridisation. For instance, in devices where information is carried from photons to magnons, this field non-reciprocity could be used (i.e., by controlling the direction of the bias field) to selectively enable or block the information exchange.

Finally, we have introduced the excitation vector fields as a way to efficiently sweep through various regimes of hybridisation. In particular, we have shown how the state of polarisation can dramatically affect spin precession inside the cavity resonator. This is particularly relevant, not only from a fundamental point of view but also practically, as understanding the role of the cavity excitation fields in governing the coupling and, consequently, the formation of cavity magnon-polaritons is crucial for effectively engineering and optimising cavity magnonic devices.

## Methods

### Experimental setup details

In order to obtain more complex field configurations within a microwave cavity resonator, we have employed a cavity shown in Fig. 1d featuring equal dimensions along *x* and *y* measuring 50 × 50 × 5 mm. A bias field, **H**_**0**_ along the ± *z* axis, was applied by placing the cavity with the sample between an electromagnet. The TE_120_ mode was obtained by exciting port 1 of the cavity, which has an anti-node at the centre. At this central point, the y-component of the magnetic field, *h*_*cy*_, is zero (or much smaller than the x-component). Thus, we can neglect *h*_*cy*_, and consider the excitation to be linearly polarised in the x-direction. Placing a small magnetic sample (YIG sphere of diameter 0.25 mm) at the centre of the cavity (at the anti-node of **h**_**c**_) enabled excitation of spin dynamics with a linearly polarised field. By subjecting the setup to an increasing magnetic field, the magnetic resonance frequency of the sample *ω*_0_ also increased until it closely matched the cavity resonance frequency *ω*_*c*_. In this frequency region, the magnons within the magnetic sample interact with the photons confined within the cavity, creating a connection between the two modes, resulting in observed quasi-Rabi splitting.

In order to induce other states of polarisation, we have introduced an additional coupler, labelled as port 2, into the cavity resonator. This second coupler generates an identical mode as the first coupler; however, its position is chosen such that the mode it generates is rotated by 90° relative to the first coupler. Therefore, the cavity field is no longer linearly polarised in the x-direction at the centre, as the second coupler now generates a y-component in the centre. The superposition of these fields still generates a linearly polarised excitation though. However, the direction of the overall oscillating magnetic field in the centre is now rotated by 45°. By controlling the phase and amplitude of the signal into the second coupler relative to the signal at the first coupler, the y-component of the driving field can be modified, allowing for more complex driving fields. This control effectively enables us to manipulate all components in Eq. (5), thus achieving driving fields with any desired polarisation state, such as circularly polarised light.

**Fig. 5:**
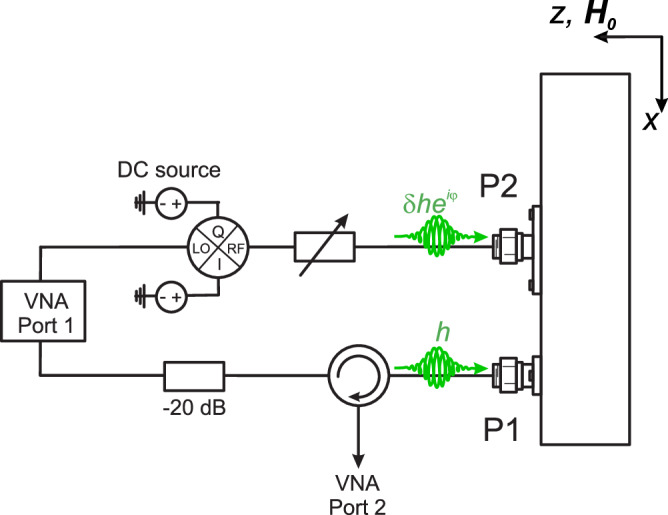
Circuit diagram for the measurements of the square cavity setup shown in Fig. 1 of the main text. The exciting signal from the VNA is split along two paths. Along the top path, the signal is phase shifted by *φ*, and attenuated by *δ* before being fed into port 2 on the cavity. A fixed attenuator was added along the bottom path, to allow for equal excitation of the two modes in the cavity, before being fed into port 1 of the cavity. Using a circulator on the bottom path, reflection from port 1 (or transmission from port 1 to port 2) is measured on a second port of the VNA.

Microwave signals were supplied by port 1 of a Rohde & Schwarz ZVA 40 vector network analyser (VNA) – a diagram of the setup can be seen in Fig. 5. The signals from the VNA were split along two paths and fed into the two ports of the square cavity [see Fig. 1d]. A static attenuator was added to one of the signal paths to ensure the initial amplitude of the two excitation signals were approximately equal. Along the signal pathway, a circulator was also added. The circulator sent signal excitation to the cavity, but signals reflected from or transmitted through the cavity were sent to port 2 of the VNA. To control the relative phase between the two cavity ports, *φ*, required for polarised excitation, a Marki IQ mixer (MLIQ0416L) was added to the other signal path. The IQ mixer was computer-controlled using a custom voltage source with built-in digital to analogue conversion. For the frequencies of interest, a map of signal phase and amplitude dependence on DC voltage was generated (See Supplemental Material D). This map was used to calibrate the IQ mixer output. To tune the relative power excitation applied to the cavity ports, *δ*, an LDA-203B Digital Attenuator was used.

In the experiment, the tones are coupled even in the absence of the YIG sample; there is a finite cross-talk. This cross-talk together with the cross-coupling is always present and leads to small deviations from the theory. However, these effects are negligible for the sample size used here and do not affect the understanding of the observed phenomena and general applicability of the theory. More details on the effect of the sample size are given in Supplemental Material F.

## Supplementary information

Supplementary Information

## Data Availability

All experimental data presented in this study is available at 10.5525/gla.researchdata.1792.
