# Supplementary material for: The role of excitation vector fields and all-polarisation state control in cavity magnonics
Source: Npj Spintron. 2024 Dec 4;2(1):59. doi: 10.1038/s44306-024-00062-z (PMC11618086; doi:10.1038/s44306-024-00062-z)
Supplement: Supplementary file 1 — Supplementary Information [file 44306_2024_62_MOESM1_ESM.pdf]

Supplementary Information for:
The role of excitation vector fields and all-polarisation state control of cavity magnonics

Alban Joseph[1,*], Jayakrishnan M. P. Nair[2], Mawgan A. Smith[1], Rory Holland[1], Luke J. McLellan[1], Isabella Boventer[3], Tim Wolz[4], Dmytro A. Bozhko[5], Benedetta Flebus[2], Martin P. Weides[1], and Rair Macêdo[1]

[1]James Watt School of Engineering, Electronics & Nanoscale Engineering Division, University of Glasgow, Glasgow G12 8QQ, United Kingdom
[2]Department of Physics, Boston College,140 Commonwealth Avenue,Chestnut Hill, MA 02467 [3]Unite Mixte de Physique CNRS, Thales, University Paris-Sud, Universite Paris-Saclay, F-91767 Palaiseau, France
[4]Institute of Physics, Karlsruhe Institute of Technology, 76131 Karlsruhe, Germany
[5]University of Colorado Colorado Springs US
*a.joseph.2@research.gla.ac.uk

Supplementary Note A: Perturbation Theory

Maxwell's equations can be used to obtain the following relation to estimate the shift in the cavity resonance frequency ω_c due to a small perturbation[27]:

$$\frac{\omega-\omega_c}{\omega_c} = -\frac{\int_{\delta v} \mu_0 [\overleftrightarrow{\chi}_m(\omega)\cdot \mathbf{h_c}]\cdot \mathbf{h_c^*} \mathrm{d}v}{2\int_v \mu_0 |\mathbf{h_c}|^2 \mathrm{d}v} \tag{S1}$$

At the sample position, if we write the a cavity excitation vector field in its most general form of $\mathbf{h_c} = (\hat{\mathbf{x}} + \hat{\mathbf{y}}\delta e^{i\varphi})h$ Eq. (S1) can be also generalised and rewritten as:

$$\frac{\omega-\omega_c}{\omega_c} = -\frac{\mu_0 \int_{\delta v} [\chi_a |h|^2 + \chi_a \delta^2 |h|^2 + i\chi_b (h^* \delta h e^{i\varphi} - h\delta h^* e^{-i\varphi})]\mathrm{d}v}{2\int_v \mu_0 |\mathbf{h_c}|^2 \mathrm{d}v}$$

or simply:

$$\frac{\omega-\omega_c}{\omega_c} = -\Big[\chi_a(1+\delta^2) - 2\chi_b \delta \sin(\varphi)\Big] \frac{\int_{\delta v} \mu_0 |h|^2 \mathrm{d}v}{2\int_v \mu_0 |\mathbf{h_c}|^2 \mathrm{d}v}. \tag{S2}$$

For the simple case of a ferromagnet, we can substitute the components of $\overleftrightarrow{\chi}_m(\omega)$ into Eq. (S2) to obtain:

$$\frac{\omega-\omega_c}{\omega_c} = -\frac{\omega_0\omega_m}{\omega_0^2-\omega^2}\Big[(1+\delta^2) - 2\frac{\omega}{\omega_0}\delta \sin(\varphi)\Big] \frac{\int_{\delta v} \mu_0 |h|^2 \mathrm{d}v}{2\int_v \mu_0 |\mathbf{h_c}|^2 \mathrm{d}v}$$

In the limit close to $\omega = \omega_0$ we obtain:

$$\frac{\omega-\omega_c}{\omega_c} = -\frac{\omega_0\omega_m}{\omega_0^2-\omega^2}\Big[1+\delta^2 - 2\delta \sin(\varphi)\Big] \frac{\int_{\delta v} \mu_0 |h|^2 \mathrm{d}v}{2\int_v \mu_0 |\mathbf{h_c}|^2 \mathrm{d}v}$$

or simply:

$$\frac{\omega-\omega_c}{\omega_c} = -\frac{\omega_0\omega_m}{\omega_0^2-\omega^2}\frac{W_p}{W_c}, \tag{S3}$$

Where $W_p = [1+\delta^2+2\delta\sin\varphi]\int_{\delta v}\mu_0|h_c|^2\mathrm{d}v$ is the magnetic energy at the sample position and $W_c = 2\int_v \mu_0|\mathbf{h_c}|^2\mathrm{d}v$ defines the total cavity energy, both measured in Joules. These quantities do not require experimental input and can be estimated using numeric solvers (such as COMSOL multiphysics) for any type of resonators, including 2D integrated devices.[27] Here, however, as we have a simple, rectangular resonator, an analytical expression for W_c can also be derived as follows:

$$W_c = 2\int_v \mu_0 |\mathbf{h_c}|^2 \mathrm{d}v$$

$$W_c = 2\mu_0 \int_0^a \int_0^b \int_0^c \mathbf{h_c^*}\cdot \mathbf{h_c}\,\mathrm{d}x\,\mathrm{d}y\,\mathrm{d}z$$

where a, b and c are the x, y and z dimensions of the cavity, respectively. Since the cavity mode is a superposition of the TE_{120} and TE_{210} mode, the oscillating magnetic field in the cavity can be written as

$$\mathbf{h_c} = \begin{bmatrix} h_{x120} \\ h_{y120} \end{bmatrix} + \begin{bmatrix} h_{x210} \\ h_{y210} \end{bmatrix} \delta e^{i\varphi}$$

We only consider the x and y components here since no magnetic field component exists in the z direction for both these modes.

Therefore,

$$\mathbf{h_c^*}\cdot\mathbf{h_c} = |h_{x120}|^2 + h_{x120}h_{x210}^*\delta e^{-i\varphi} + h_{x210}h_{x120}^*\delta e^{i\varphi} + \delta^2|h_{x210}|^2 + |h_{y120}|^2 + h_{y120}h_{y210}^*\delta e^{-i\varphi} + h_{y210}h_{y120}^*\delta e^{i\varphi} + \delta^2|h_{y210}|^2$$

The magnetic field distribution for a TE$_{mnl}$ mode in a rectangular cavity can be written as

$$h_{cx} = iA\frac{\kappa_{0y}}{\omega_c\mu_0}\sin\kappa_{0x}x\cos\kappa_{0y}y \tag{S4}$$

$$h_{cy} = -iA\frac{\kappa_{0x}}{\omega_c\mu_0}\cos\kappa_{0x}x\sin\kappa_{0y}y \tag{S5}$$

where $\kappa_{0x} = m\pi/a, \kappa_{0y} = n\pi/b$ and A is an amplitude constant.

With these definitions:

$$\int_0^a\int_0^b h_{x210}h_{x120}^*\,\mathrm{d}x\,\mathrm{d}y = \int_0^a\int_0^b h_{x120}h_{x210}^*\,\mathrm{d}x\,\mathrm{d}y = \int_0^a\int_0^b h_{y210}h_{y120}^*\,\mathrm{d}x\,\mathrm{d}y = \int_0^a\int_0^b h_{y120}h_{y210}^*\,\mathrm{d}x\,\mathrm{d}y = 0$$

Therefore W_c can be written as

$$W_c = 2\mu_0\int_0^a\int_0^b\int_0^c |h_{x120}|^2 + \delta^2|h_{x210}|^2 + |h_{y120}|^2 + \delta^2|h_{y210}|^2\,\mathrm{d}x\,\mathrm{d}y\,\mathrm{d}z$$

By substituting field distributions we get

$$W_c = 2\mu_0\int_0^c \frac{1}{\mu_0^2\omega_c^2}\frac{5\pi^2(1+\delta^2)}{4}\,\mathrm{d}z$$

$$W_c = \frac{5\pi^2\left(1+\delta^2\right)c}{2\mu_0\omega_c^2}$$

We can solve Eq. (S3) for ω close to both ω_c and ω_0, and obtain an analytical expression for the eigenfrequencies. Since we are able to include the new terms due to δ and φ into W_p and W_c, our resulting equation remain the same as that obtained in previous work[27]:

$$\omega_{a,b} = \frac{1}{2}\left[\omega_c + \omega_0 \pm \sqrt{(\omega_c-\omega_0)^2 + 2\omega_c\omega_m\frac{W_p}{W_c}}\right], \tag{S6}$$

where, ω_a and ω_b are the eigenfrequencies of the cavity-magnon hybrid system.

The macroscopic coupling strength, g, is related to the width of the splitting between the eigenfrequencies at $\omega_0 = \omega_c$, where $2g = |\omega_a - \omega_b| = \Delta\omega$. Therefore, we can then take the eigenfrequencies of the system at $\omega_0 = \omega_c$ from Eq. (S6) which take the form:

$$\omega_{a,b} = \omega_c \pm \frac{1}{2}\sqrt{2\omega_c\omega_m\frac{W_p}{W_c}} \tag{S7}$$

We can, therefore use this result to calculate the size of the Rabi splitting:

$$\Delta\omega = \omega_a - \omega_b = \sqrt{2\omega_c\omega_m\frac{W_p}{W_c}}. \tag{S8}$$

Figure S1 presents the magnetisation precession dynamics and the hybridisation behaviour observed across the entire range of excitation field polarisations and bias field orientations. The green arrows in panels (a) and (b) depict the polarisation state of the excitation field for various values of δ and φ. The red arrows in (c) and (d) correspond to the subsequent magnetisation precession in the YIG sample, calculated from the Landau-Lifshitz-Gilbert (LLG) equations, for bias fields along $+\hat{\mathbf{z}}$ in (c) and $-\hat{\mathbf{z}}$ in (d), respectively. The calculated values for $\Delta\omega/2\pi$ from Eq. (S8) are presented in the heat maps of Fig. S1 for a bias field of $+\hat{\mathbf{z}}H_0$ in (e) and $-\hat{\mathbf{z}}H_0$ in (f). We see good agreement between these theoretical predictions and the experimental data presented in Fig. 4.

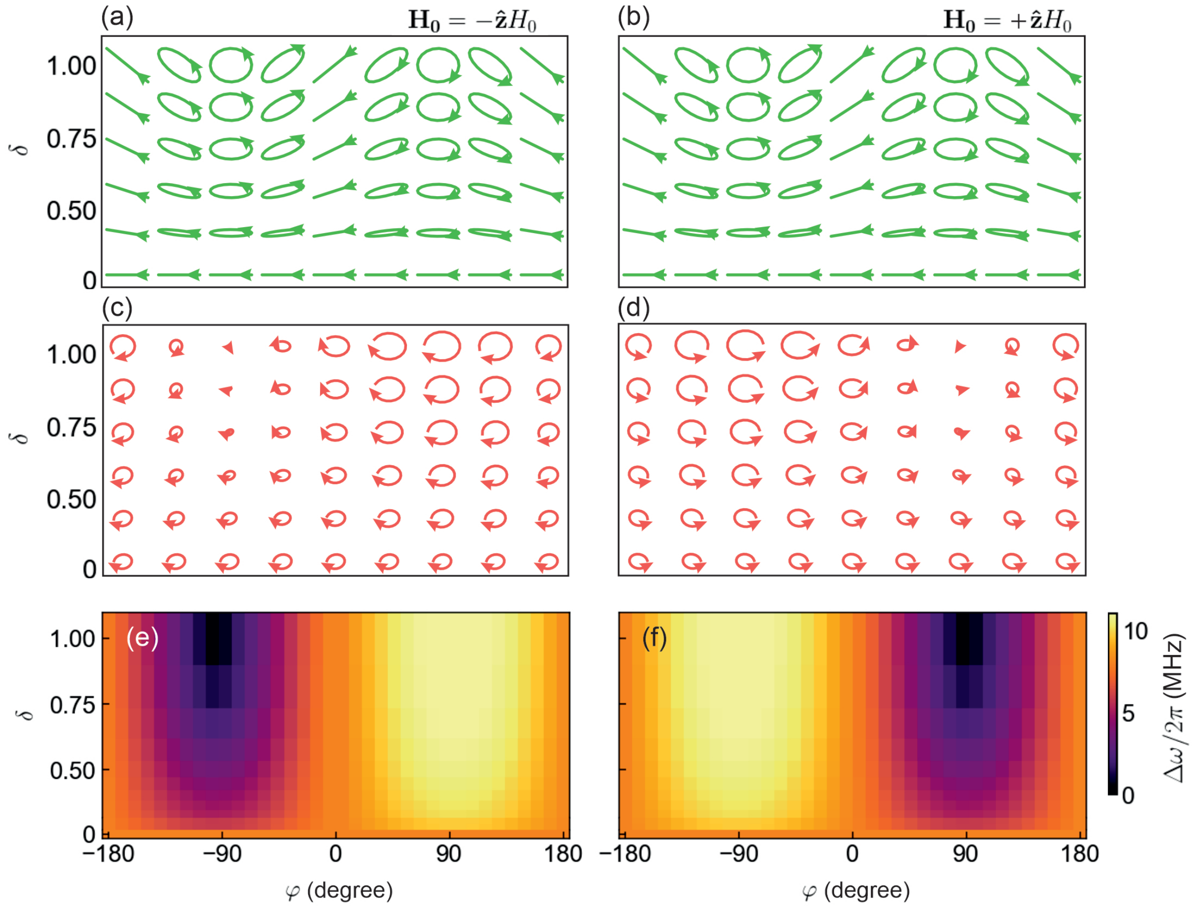

Fig S1. (a) and (b) Diagrams illustrating the polarisation of the driving field for different values of δ and φ for a bias field of $+\hat{\mathbf{z}}H_0$ and $-\hat{\mathbf{z}}H_0$, respectively. (c) and (d) show the subsequent spin precession resulting from the excitation fields and bias fields in (a) and (b), respectively, calculated using the Landau-Lifshitz-Gilbert (LLG) equations. Heat maps summarising the width of the quasi-Rabi splitting, $\Delta\omega/2\pi$, as a function of δ and φ, calculated using perturbation theory, are presented in (e) for a bias field of $+\hat{\mathbf{z}}H_0$ and in (f) for a bias field of $-\hat{\mathbf{z}}H_0$.

Special case: Circularly polarised excitation vector field

Now if we introduce a left or right circularly polarised excitation vector field at the sample position by plugging $\delta = 1, \varphi = \pm 90$ into Eq. (S2) we obtain:

$$\frac{\omega - \omega_c}{\omega_c} = -2\left[\chi_a \mp \chi_b\right] \frac{\int_{\delta v} |h|^2 \mathrm{d}v}{2\int_v |\mathbf{h_c}|^2 \mathrm{d}v} \tag{S9}$$

which can be rewritten as:

$$\frac{\omega - \omega_c}{\omega_c} = -2\left[\frac{\omega_0 \omega_m}{\omega_0^2 - \omega^2} \mp \frac{\omega \omega_m}{\omega_0^2 - \omega^2}\right] \frac{\int_{\delta v} |h|^2 \mathrm{d}v}{2\int_v |\mathbf{h_c}|^2 \mathrm{d}v} = -\frac{\omega_m}{(\omega_0 \pm \omega)} \frac{\int_{\delta v} |h|^2 \mathrm{d}v}{\int_v |\mathbf{h_c}|^2 \mathrm{d}v}. \tag{S10}$$

This result can also be obtained by directly combining Eq. (2) directly into Eq. (S1) to obtain:

$$\frac{\omega - \omega_c}{\omega_c} = -\frac{\omega_m}{\omega_0 \pm \omega} \frac{\int_{\delta v} |\mathbf{h}^{\mp}|^2 \mathrm{d}v}{2\int_v |\mathbf{h_c}|^2 \mathrm{d}v}. \tag{S11}$$

Supplementary Note B: Quantisation of Cavity Fields and Input-Output Theory

In this section, we provide additional detail on the quantised formalism for microwave cavities and their response through reflection.

Input-Output Theory of the Cavity and Reflection

The interplay between the incoming and outgoing travelling wave excitations on a cavity can be then used to find the scattering matrix elements for reflection and transmission coefficients. The cavity modes interact with the continuum of electromagnetic modes outside the cavity. The interaction between the cavity mode a and the continuum can be modelled as[60]

$$\frac{H}{\hbar} = \omega_a a^\dagger a + \int_{-\infty}^{\infty} d\omega[\omega b^\dagger(\omega) b(\omega)] + i\int_{-\infty}^{\infty} d\omega g(\omega)[a^\dagger b(\omega) - a b^\dagger(\omega)], \tag{S12}$$

where $b(\omega)(b^\dagger(\omega))$ is the annihilation (creation) operator of the continuum of electromagnetic modes satisfying $[b(\omega), b^\dagger(\omega')] = \delta(\omega-\omega')$, while $g(\omega)$ parameterizes strength of interaction between the cavity mode and the continuum. The Heisenberg equation of motion (EOM) of the mode a is given by

$$\dot{a} = -i\omega a - \int_{-\infty}^{\infty} g(\omega) e^{-i\omega(t-t')} b(t') dt'. \tag{S13}$$

By the same token, the EOM for the b modes can be obtained, upon formal integration, as

$$b(\omega,t) = e^{-i\omega(t-t_{-\infty})} b(\omega, t_{-\infty}) + g(\omega)\int_{t_{-\infty}}^{t} e^{-i\omega(t-t')} a(t') dt', \tag{S14}$$

for $t > t_-\infty$ and

$$b(\omega,t) = e^{-i\omega(t-t_{\infty})} b(\omega, t_{\infty}) - g(\omega)\int_{t}^{t_\infty} e^{-i\omega(t-t')} a(t') dt', \tag{S15}$$

for $t < t_\infty$. Substituting Eq. (S14) and Eq. (S15) into Eq. (S13) and defining

$$a_{in}(t) = -\frac{1}{\sqrt{2\pi}}\int_{-\infty}^{\infty} d\omega e^{-i\omega(t-t_{-\infty})} b(\omega, t_{-\infty}),$$
$$a_{out}(t) = \frac{1}{\sqrt{2\pi}}\int_{-\infty}^{\infty} d\omega e^{-i\omega(t-t_{\infty})} b(\omega, t_{\infty}), \tag{S16}$$

we obtain

$$\dot{a} = -i\omega a - \kappa a + \sqrt{2\kappa} a_{in}(t), \tag{S17}$$
$$\dot{a} = -i\omega a - \kappa a - \sqrt{2\kappa} a_{out}(t), \tag{S18}$$

where we have used the Markov approximation $g^2(\omega) = \kappa/\pi$. It follows from Eq. (S17) that

$$a_{in} + a_{out} = \sqrt{2\kappa} a. \tag{S19}$$

The Eq. (S19) is identical to the Eq. (13) in the main text.

Quantisation of the cavity fields using two ports

While in the main text we have chosen the present the quantised cavity fields as a single effective polarised mode, in the context of our experiment, the cavity is driven by two ports, which excite orthogonally polarised modes $a_{\vec{k}x}$ and $a_{\vec{k}y}$ at the same frequency ω_c. The dynamics of these modes in the rotating frame of the drive are described by

$$\begin{pmatrix} \dot{a}_{\vec{k}x} \\ \dot{a}_{\vec{k}y} \end{pmatrix} = M \begin{pmatrix} a_{\vec{k}x} \\ a_{\vec{k}y} \end{pmatrix} + \begin{pmatrix} \varepsilon_{p_x} \\ \varepsilon_{p_y} \end{pmatrix} + \begin{pmatrix} a^{in}_{\vec{k}x} \\ a^{in}_{\vec{k}y} \end{pmatrix}, \tag{S20}$$

where $\varepsilon_{p_s} = \sqrt{\frac{2\kappa D_{p_s}}{\hbar\omega_c}}$ are the amplitudes of the external drive with drive powers D_{p_s}, $s \in \{x,y\}$, $M = \begin{pmatrix} -i\Delta_d - \kappa & 0 \\ 0 & -i\Delta_d - \kappa \end{pmatrix}$, κ represents the cavity decay rate, and $a^{in}_{\vec{k}(x,y)}$ are quantum Langevin noise with $\langle a^{in}_{\vec{k}(x,y)} \rangle = 0$. The parameter $\Delta_d = \omega_{\vec{k}x} - \omega_p$, and for simplicity, we assume $\omega_{\vec{k}_x} = \omega_{\vec{k}_y}$.

In the long-time limit, the steady-state amplitudes of the modes are given by $\langle a_{\vec{k}x}\rangle = M_{11}^{-1}\varepsilon_{d_x}$ and $\langle a_{\vec{k}y}\rangle = M_{22}^{-1}\varepsilon_{d_y}$. Noting that $M_{22}^{-1} = M_{11}^{-1}$ and considering the experimental condition $\varepsilon_{d_x}/\varepsilon_{d_y} = \delta e^{i\varphi}$, the time-independent part of the vector potential can be expressed as

$$\vec{A} = \sqrt{\frac{2\pi\hbar c^2}{\omega_k v}}\left(\hat{x}+\delta e^{i\varphi}\hat{y}\right)M_{11}^{-1}f(\vec{k},\vec{r}) + \text{h.c.}$$

This result indicates that the two-port drive effectively prepares the cavity in a superposition of the two modes $a_{\vec{k}(x,y)}$ with polarisation $\hat{x}+\delta e^{i\varphi}\hat{y}$. Therefore, the cavity can be described by a single effective mode a, which is a superposition of $a_{\vec{k}(x,y)}$. Thus, yielding the same result as that obtained when considering a single, polarised mode to start with.

We note that the reflection spectroscopy performed around the steady state probes the effective mode a. Therefore, since this result is effectively the same (whether the starting point is a superposition of two-mode drives with distinct polarisations or a single polarised mode) it does not require any modification to input-output theory shown above in terms of a. The interaction of this effective mode a with the continuum of electromagnetic modes in the cavity is consistent with the calculations provided in this supplementary note.

Supplementary Note C: Phase Data

The S_{11} parameter is a complex quantity, so an alternative approach to determining the coupling from the amplitude data, as done in Fig. 2, is to infer the same information from the phase data which is given in Fig. S2. We can see that the phase closely matched the data shown in the main article Fig. 2 where a sharp change in phase corresponds to a dip in amplitude. Thus, two branches are seen for the cases where the chirality of the polarised excitation vector field matches that of the magnon modes (the distance between both branches of phase change at $\omega_c = \omega_0$ can be used to obtain $\Delta\omega$ and therefore g) and a single, flat region of sharp change in phase for the cases where the chirality of the magnon mode is opposite to that of the excitation polarisation corresponding to the frequency of the cavity mode ω_c.

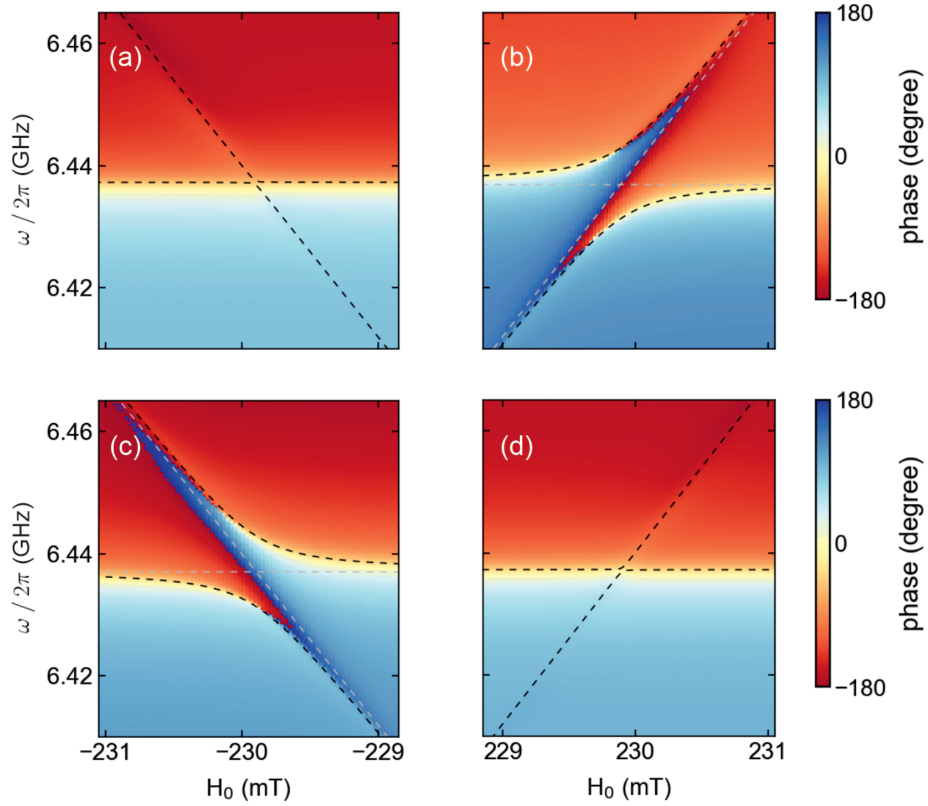

Fig S2. Experimental S_{11} phase spectra and perturbation theory (dashed black lines) of the Rabi splitting close to $\omega_0 = \omega_c$ for (a) $\mathbf{H_{eff}} = -\hat{\mathbf{z}}H_0 + \mathbf{h}^+$; (b) $\mathbf{H_{eff}} = \hat{\mathbf{z}}H_0 + \mathbf{h}^+$; (c) $\mathbf{H_{eff}} = -\hat{\mathbf{z}}H_0 + \mathbf{h}^-$ and (d) $\mathbf{H_{eff}} = \hat{\mathbf{z}}H_0 + \mathbf{h}^-$. The dashed grey lines show the cavity mode and Kittel mode.

Supplementary Note D: Experimental set-up optimisation and calibration

IQ Mixer – Calibration

To control the phase difference between the two input signals using the IQ mixer, a map of signal phase and amplitude dependence on DC voltage was generated. The phase at ports 1 and 2 was measured and the maps shown in Fig. S3 were then used to set the phase at port 2 to the desired value. The phase that corresponded to the least attenuation of the signal was selected, i.e. RF/LO ≈ 1.

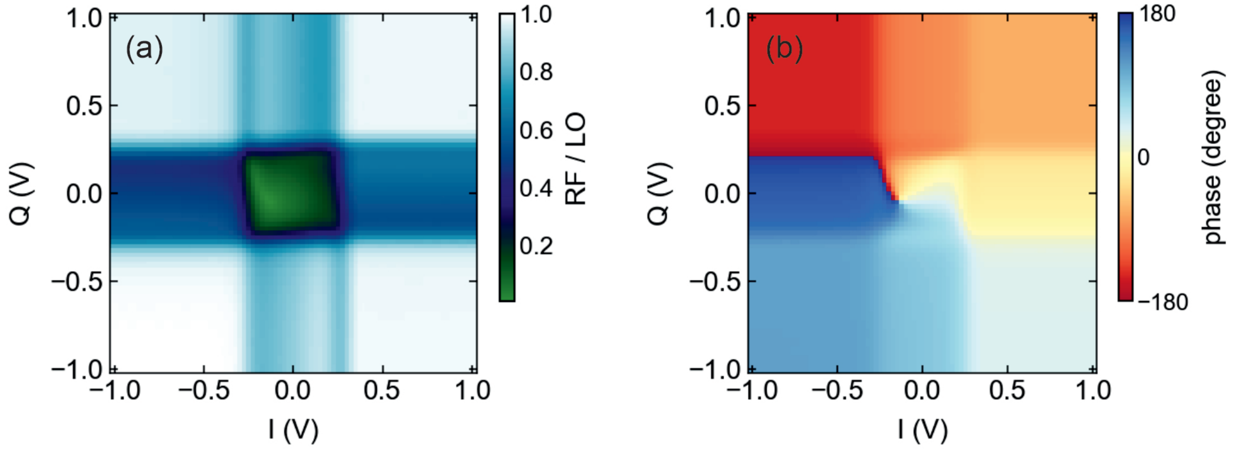

Fig S3. IQ mixer (a) amplitude response and (b) phase response to DC voltage input

Extraction of Cavity-Photon and Magnon Dissipation

In addition to controlling the phase difference between the two input signals, we also calibrate the amplitude ratio between them both. For this, we place an attenuator to the path of one of the signals in order to ensure both initial amplitudes were nearly the same; making $\delta = 1$. As discussed in the theory section, our assumption is that both inputs create identical modes so that it can be considered as a single effective mode a, and therefore calibrating $\delta = 1$ is the first step to achieving this. Once this is complete, and before starting a measurement, we tune the coupling to each mode until we obtain equal dissipation in both modes (or near equal, as limited by experimental conditions) which can be done by fitting for the HWHM far from magnetic resonance. Once this is obtained, data can be gathered. From this point forward, we can then treat the experiment as having a single mode, and parameters such as the total dissipation can be used

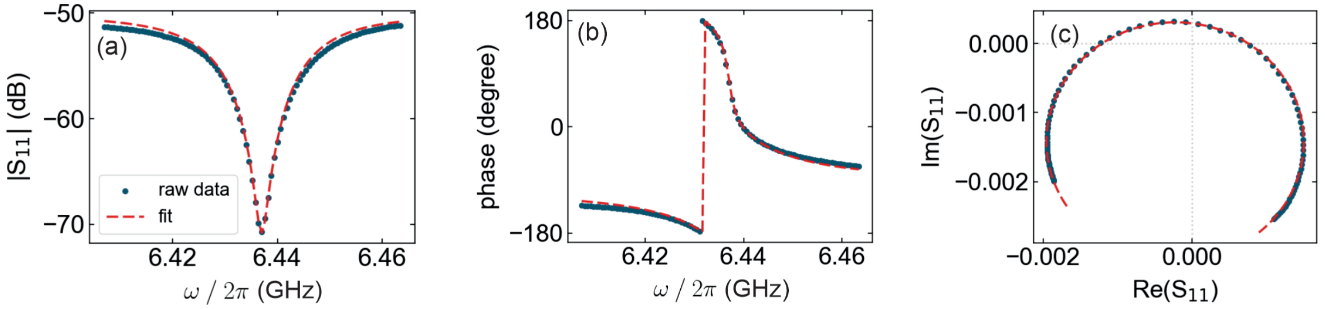

Fig S4. Circle fit analysis of the effective cavity mode for an arbitrary phase, φ. (a) shows the S_{11} amplitude, (b) shows the S_{11} phase, and (c) presents the circle fit of the complex S_{11} data. The blue points indicate the raw data, while the red dashed line shows the corresponding fit.

To extract the intrinsic and coupling quality factors, we employed the circle fit method[61] for the combined effective mode, shown in Fig. S4.The loaded quality factor Q_l was then used to extract κ using the relation $\kappa = f_r/2Q_l$. Both resonance frequency and quality factors remain largely the same throughout our measurement. However, due to

experimental limitations, slight deviations were observed, resulting in minor shifts in the cavity resonance and quality factors as the phase, φ, was varied (as shown in Fig. S5). To obtain the overall cavity resonance ω_c and dissipation κ, we averaged these values over all phases.

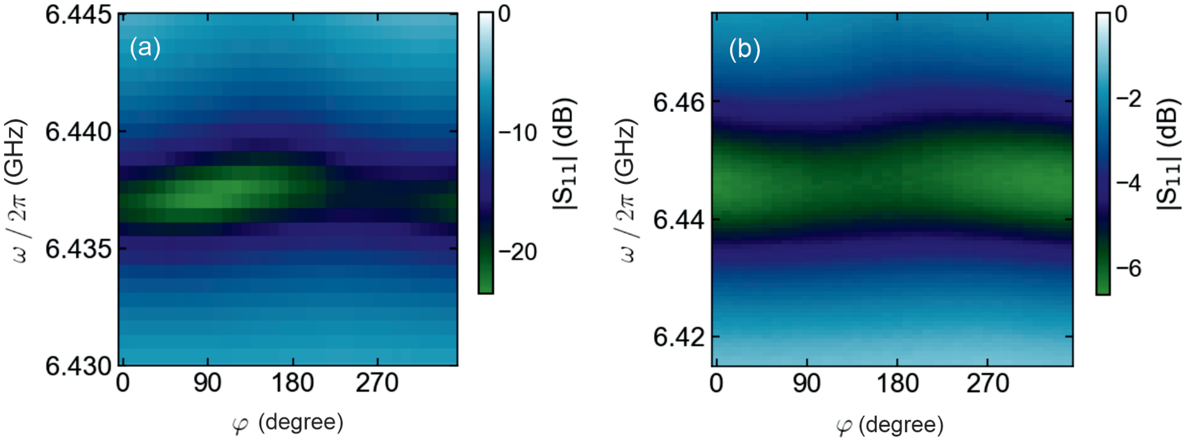

Fig S5. Empty cavity mode as a function of phase for the experiment run using (a) the 0.25 mm diameter YIG sphere and (b) the 0.5 mm diameter YIG sphere.

Based on our circle fit analysis, we derive the following quality factors for the effective mode after averaging across all phases:

- Resonant frequency, $f_r = 6.43772$ GHz
- Intrinsic quality factor, $Q_i = 530.49$
- Coupling quality factor, $Q_c = 448.01$
- Loaded quality factor, $Q_l = 242.89$

From these measurements, we therefore extract $\kappa = 13.252$ MHz, which is the value used in our plots throughout this work. For completeness, we also provide below the results for excitation with one port only, i.e. for $\delta = 0$. We find the following parameters:

- Resonant frequency, $f_r = 6.4382$ GHz
- Intrinsic quality factor, $Q_i = 524.6$
- Coupling quality factor, $Q_c = 444.5$
- Loaded quality factor, $Q_l = 240.6$

From this, we extract $\kappa = 13.378$ MHz. Note that these are nearly identical to the effective mode (when both ports are on). Thus. reassuring our premise that while driven by two inputs our system can indeed be treated as a single, polarised cavity mode.

The magnon dissipation, or relaxation rate η, was determined by fitting a Lorentzian curve to the magnon mode far from the cavity resonance. The value of η was calculated from the HWHM, or linewidth ΔH, of the fitted Lorentzian using the expression using the equation $\eta = \gamma \Delta H$, where γ is the gyromagnetic ratio[62].

Supplementary Note E: Superposition of Two Cavity Fields

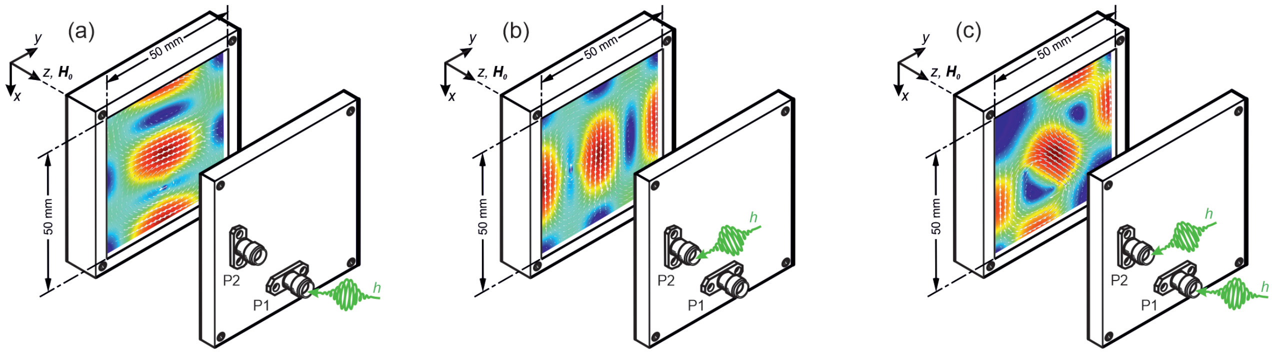

Fig S6. The cross-sectional field configuration at z = 2.5 mm generated through capacitive coupling (simulated with COMSOL) within a square cavity resonator. (a) The oscillating magnetic field when excited only through port 1 (P1), (b) the oscillating magnetic field when excited only through port 2 (P2) and (c) the oscillating magnetic field when excited equally through both port 1 (P1) and port 2 (P2).

The generation of controllable polarised excitation fields, including circular, elliptical, and linear polarisations, is achieved through the superposition of two orthogonal cavity modes. The TE_{120} mode has the field profile shown in Fig. S6(a) and was obtained by exciting port 1 of the cavity. The oscillating $\mathbf{h_c}$ intensity profile shown has an anti-node at the centre. At this central point, the $y-$component of the magnetic field, h_{cy}, is zero. Thus, we can neglect h_{cy}, and consider the excitation vector field to be linearly polarised in the $x-$direction. Port 2 generates an identical mode to port 1, however, it is placed in such a position that the fields it generates are rotated by 90°, relative to the first port – i.e, the TE_{210} mode, as shown in panel (b). This mode only has a y-component at the centre of the cavity. When exciting with both ports with equal excitation, as shown in panel (c), the cavity excitation vector fields now have an $x-$component and a $y-$component at the centre. The superposition of these fields still generates a linearly polarised excitation, but the direction of the overall oscillating magnetic field in the centre is now rotated by 45°. By controlling the phase and amplitude of the signal into the second coupler relative to the signal at the first coupler, the $y-$component of the driving field at the centre can be modified, allowing for more complex driving fields, such as circularly polarised light.

To validate our analysis, we examined the magnetic field components at the sample position (centre of the cavity) using COMSOL.

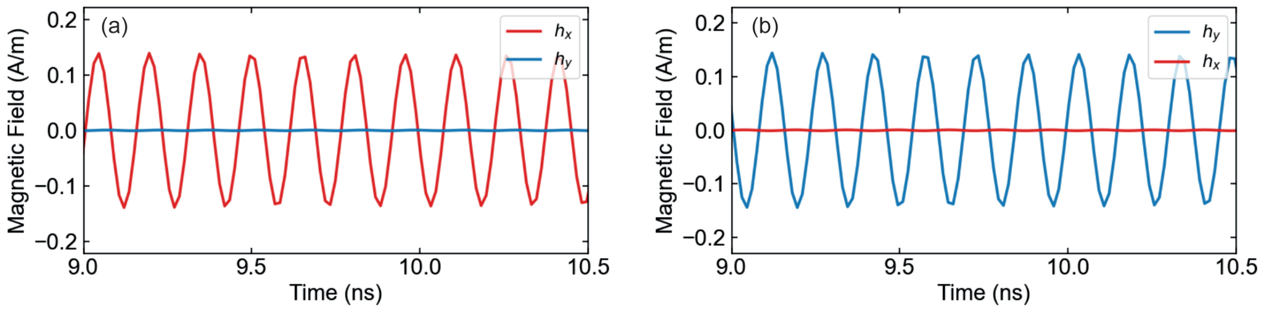

Fig S7. COMSOL simulations showing the x and y components of the magnetic field at the sample position (centre of the cavity) within the square cavity resonator for (a) the TE_{120} mode (excited by Port 1) and (b) the TE_{210} mode (excited by Port 2).

The results presented in Fig.S7 corroborate our theoretical predictions as described by Eq.(S4) and Eq. (S5). For the TE_{120} mode (excited by Port 1), we observe a dominant x-component of the magnetic field, while the y-component is negligible. Conversely, the TE_{210} mode (excited by Port 2) exhibits a dominant y-component, with a negligible x-component.

It therefore follows that by simultaneously exciting both the TE_{120} and TE_{210} modes, (i.e. by exciting at both Port 1 and Port 2 at the same time) we can generate excitation fields at the cavity's center with both x and y components.

The relative phase between these two modes allows us to achieve various polarisation states, as shown in Fig. S8.

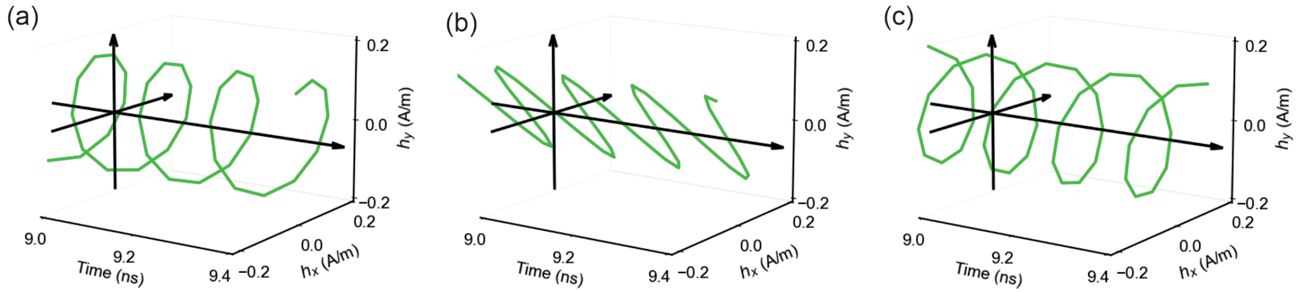

Fig S8. COMSOL simulations showing the x and y components of the magnetic field at the sample position (center of the cavity) within the square cavity resonator. The excitations applied to Port 1 and Port 2 maintain equal amplitude ratios, $\delta = 1$, while varying the phase difference: (a) $\varphi = -90^{\circ}$, (b) $\varphi = 0^{\circ}$, and (c) $\varphi = +90^{\circ}$.

By manipulating the phase difference between the two modes, φ, we can precisely control the polarisation state of the resulting field. Furthermore, adjusting the amplitude ratio, δ, between the two components provides an additional degree of freedom, allowing for even finer control over the field configuration.

Supplementary Note F: Limitation of Experimental Set Up

Introduction of transmitted energy

At higher amplitude ratios, specifically when $\delta > 1$, the experimental setup faced limitations that stopped it from operating correctly.

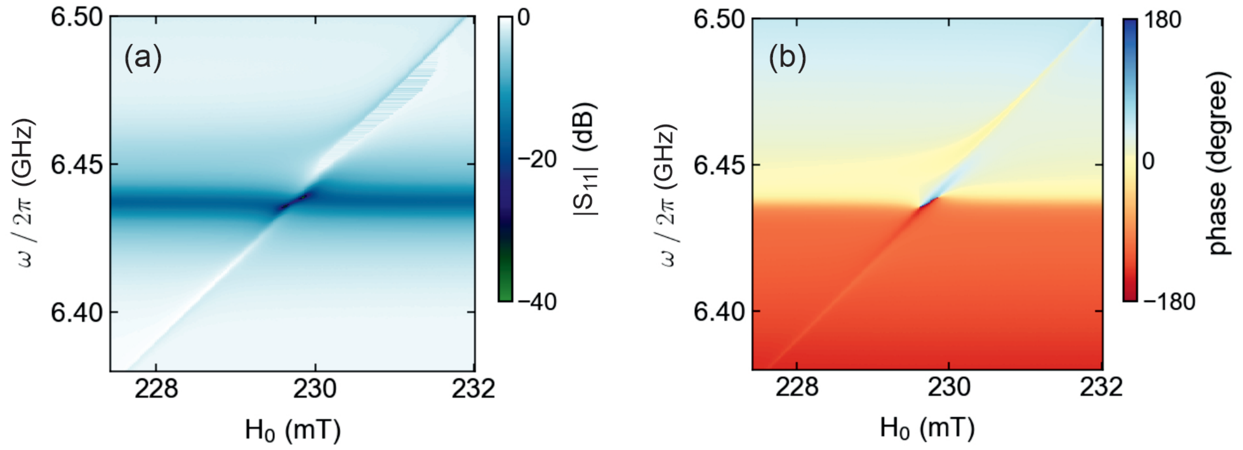

Fig S9. Experimentally measured $|S_{11}|$ amplitude data in (a) and phase data in (b) for $\delta \approx 1.3$, $\varphi \approx +90^\circ$ and $\mathbf{H_0} = +\hat{\mathbf{z}}H_0$

The reflection data presented in Fig. S9 corresponds to measurements obtained at approximately $\delta \approx 1.3$ and $\varphi \approx +90$. The observed spectrum resembles level attraction, however, it is unlikely to represent a true manifestation of this phenomenon. According to perturbation theory, level attraction is not expected to occur for any polarisation state. This is also in agreement with our quantised in-and-out model by the absence of non-hermitian terms. Instead, we interpret this observed behavior as an artefact stemming from our experimental setup.

While our experimental setup is designed to measure reflection from port 1, an amplitude ratio of $\delta > 1$ results in a higher excitation at port 2 in comparison to port 1. At these amplitude ratios, energy is transmitted from port 2 to port 1 and subsequently, the experimental conditions can no longer be considered purely reflective, but begin to include a transmission component. To confirm, we increased the amplitude ratio, eventually observing a transition in the spectrum to a pure transmission profile. Thus, we believe the appearance resembling level attraction is likely a result of the combination of reflection and transmission in the spectrum rather than an authentic manifestation of level attraction.

Consequently, this experiment is limited to $\delta \leq 1$. However, in order to investigate $\delta \geq 1$, one can simply place the circulator on the other signal path to measure the reflection from port 2 of the cavity (into port 2 of the VNA) instead. In this configuration, when port 2 exhibits higher excitation compared to port 1, i.e. when $\delta > 1$, we can still obtain a purely reflection measurement. We expect the same hybridisation behaviour for $0 \leq \delta \leq 1$ as for $1 \leq \delta < \infty$, since the excitation polarisation for both these ranges is identical. For this reason, we limited our investigation to varying δ from 1 to 0.

Size Effects

The measurements were repeated for larger samples; results for the 0.5 mm diameter YIG sphere are shown in Figs. S10–S11. From Fig. S10, it is evident that with right and left circularly polarised excitation, we were still able to sweep across coupling regimes—level repulsion and annihilation of the cavity magnon-polaritons. Similar to the case of the 0.25 mm diameter YIG sphere, we have field non-reciprocity, so this system could still find applications in information processing, such as an on/off switch.

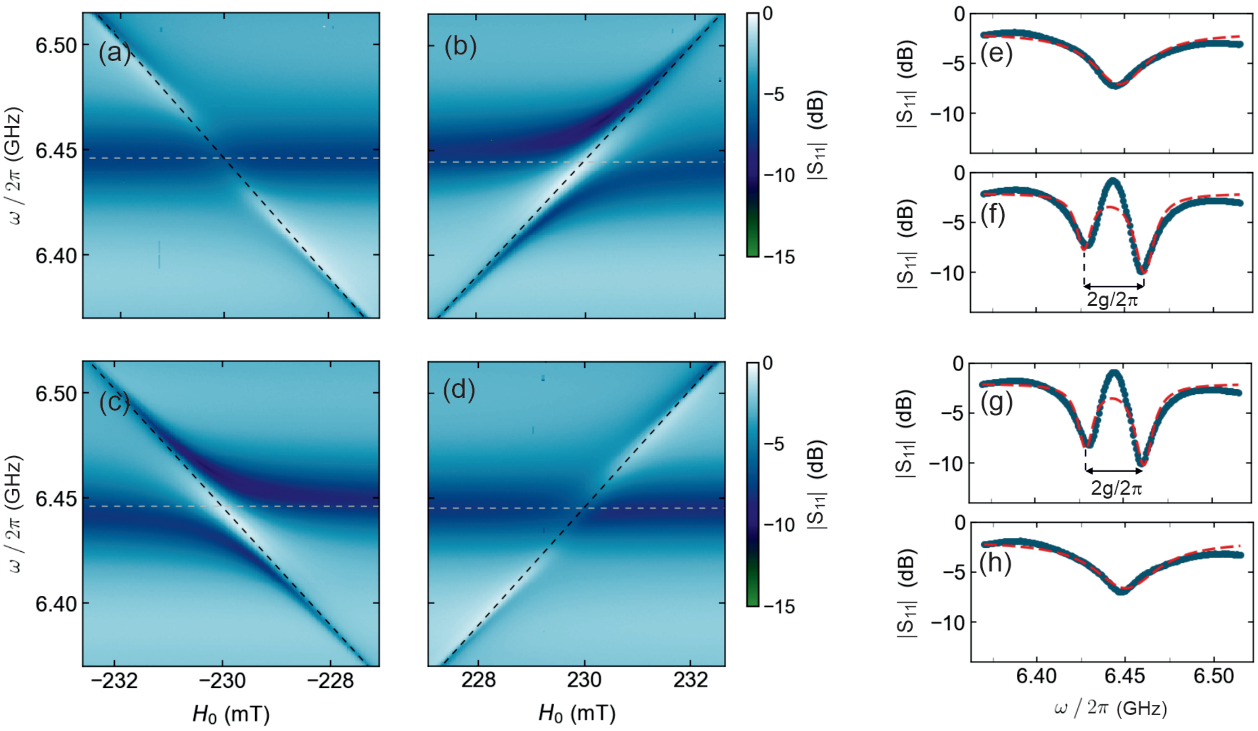

Fig S10. Experimental spectra of the Rabi splitting using a 0.5mm diameter YIG sphere for (a) $\mathbf{H_{eff}} = -\hat{\mathbf{z}}H_0 + \mathbf{h}^+$; (b) $\mathbf{H_{eff}} = \hat{\mathbf{z}}H_0 + \mathbf{h}^+$; (c) $\mathbf{H_{eff}} = -\hat{\mathbf{z}}H_0 + \mathbf{h}^-$ and (d) $\mathbf{H_{eff}} = \hat{\mathbf{z}}H_0 + \mathbf{h}^-$. The dashed lines show the cavity mode (grey) and Kittel mode (black). (e)-(h) show the corresponding $|S_{11}|$ parameter measured when $\omega_0 = \omega_c$.

Fig. S11 summarises the hybridisation behaviour under various driving conditions. Panel (a) and (b) show the effect changing the phases on port 2 relative to port 1 with an amplitude ratio of $\delta = 1$ has on the hybridisation behaviour. In comparison to Fig. 3 (the results for the 0.25 mm diameter YIG sphere), we can observe that the path loosely follows the same shape. We still see a mode crossing and mode repulsion for left- and right circularly polarised fields, with respect to the bias field $\mathbf{H_0}$, consistent with the smaller sample. However, when exciting with fields near left circular polarisation, the shape begins to deviate from the expected form. We believe this artefact emerges as a result of the larger sample. Since it is larger, the sample no longer interacts with the superposition of cavity modes as a single mode but rather as two degenerate modes. As a result, the sample was coupling more strongly to one mode compared to the other, leading to the observed shape distortion.

Panels (c) and (d) can be compared to Fig. 4. Again, the shape is somewhat similar but not exactly the same. The most noticeable difference was that the enhanced coupling region (near the right circularly polarised excitation) was broader, and the area showing decreased coupling (near the left circularly polarised excitation) was narrower. Additionally, the enhancement in coupling for right-circularly polarised excitation compared to linearly polarised excitation was not as significant as observed with the smaller sample. With the smaller sample, we saw an enhancement in the coupling strength by a factor of $\sqrt{2}$, whereas for the 0.5 mm diameter YIG sphere, the enhancement in coupling strength was not as significant (increased from 12.5 MHz when exciting the sample with a single tone, for $\delta = 0$, to 15.5 MHz for right-circularly polarised excitation).

The experimental results obtained using the 1mm diameter YIG sphere revealed significant differences compared to smaller samples. These findings suggest that the sample was no longer coupled to a single superimposed tone. Instead, it exhibited distinct coupling behaviours for each individual tone. For left circularly polarised excitation fields

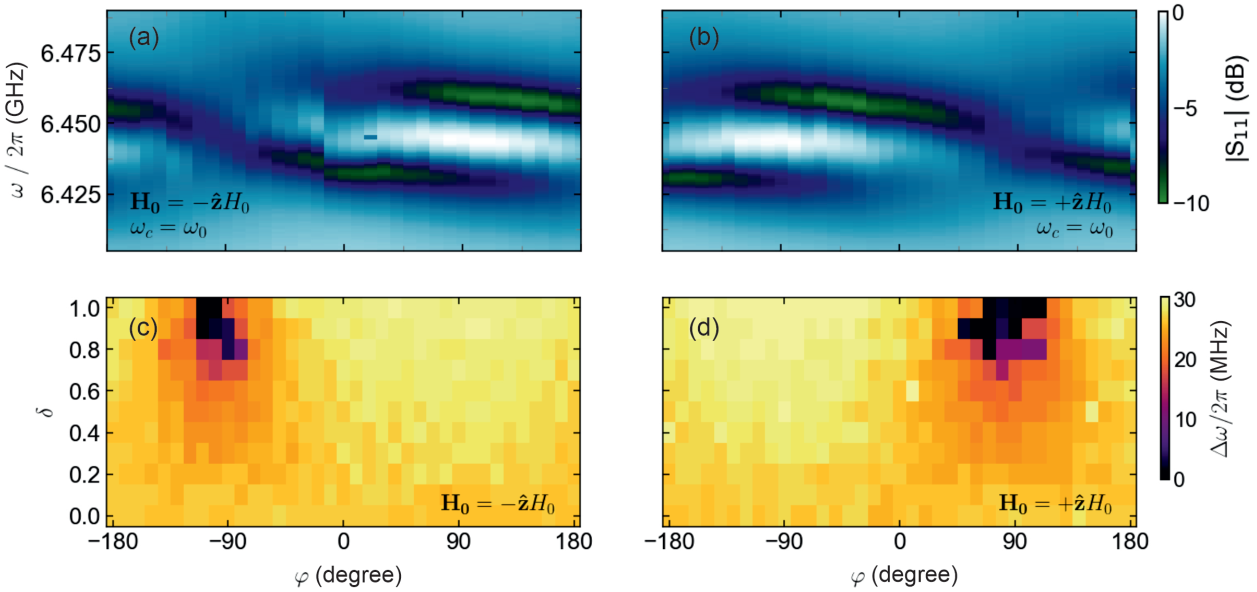

Fig S11. The experimental amplitudes of the $|S_{11}|$ parameter for various values of φ when $\omega_0 = \omega_c$ using a 0.5mm diameter YIG sphere is shown for a bias field of $+\hat{\mathbf{z}}H_0$ in (a) and $-\hat{\mathbf{z}}H_0$ in (b). The experimentally measured quasi-Rabi splitting, $\Delta\omega/2\pi$, for various δ and φ using a 0.5mm diameter YIG sphere for the same bias fields are shown in (c) and (d) respectively.

with respect to the bias field, we observed a level crossing. However, the spectra also displayed a component of level repulsion. This type of spectral behaviour featuring the appearance of three eigenmodes has been seen previously in the literature[42,43,63]. It's important to note that the foundation of this experiment, and on cavity magnon-polariton studies in general, relies on the sample minimally disturbing the cavity fields. However, with the larger 1mm sphere, the sample's influence became more pronounced. This increased interaction allowed the sample to mediate crosstalk between the two modes, ultimately giving rise to the observed complex coupling behaviour. The datasets for all sample sizes is presented below.

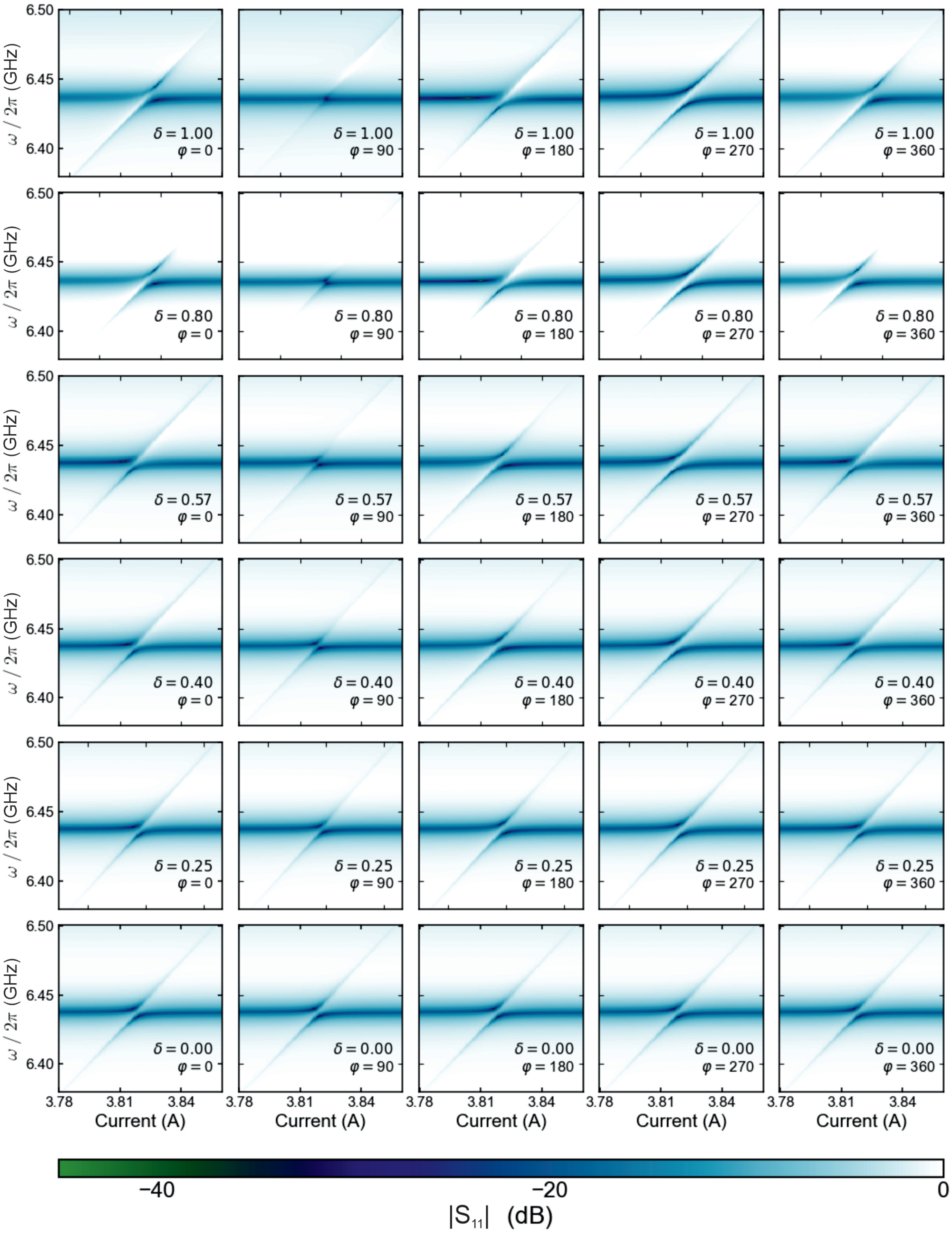

Fig S12. Experimental data using 0.25mm diameter YIG sphere for $\mathbf{H_0} = +\hat{\mathbf{z}}H_0$.

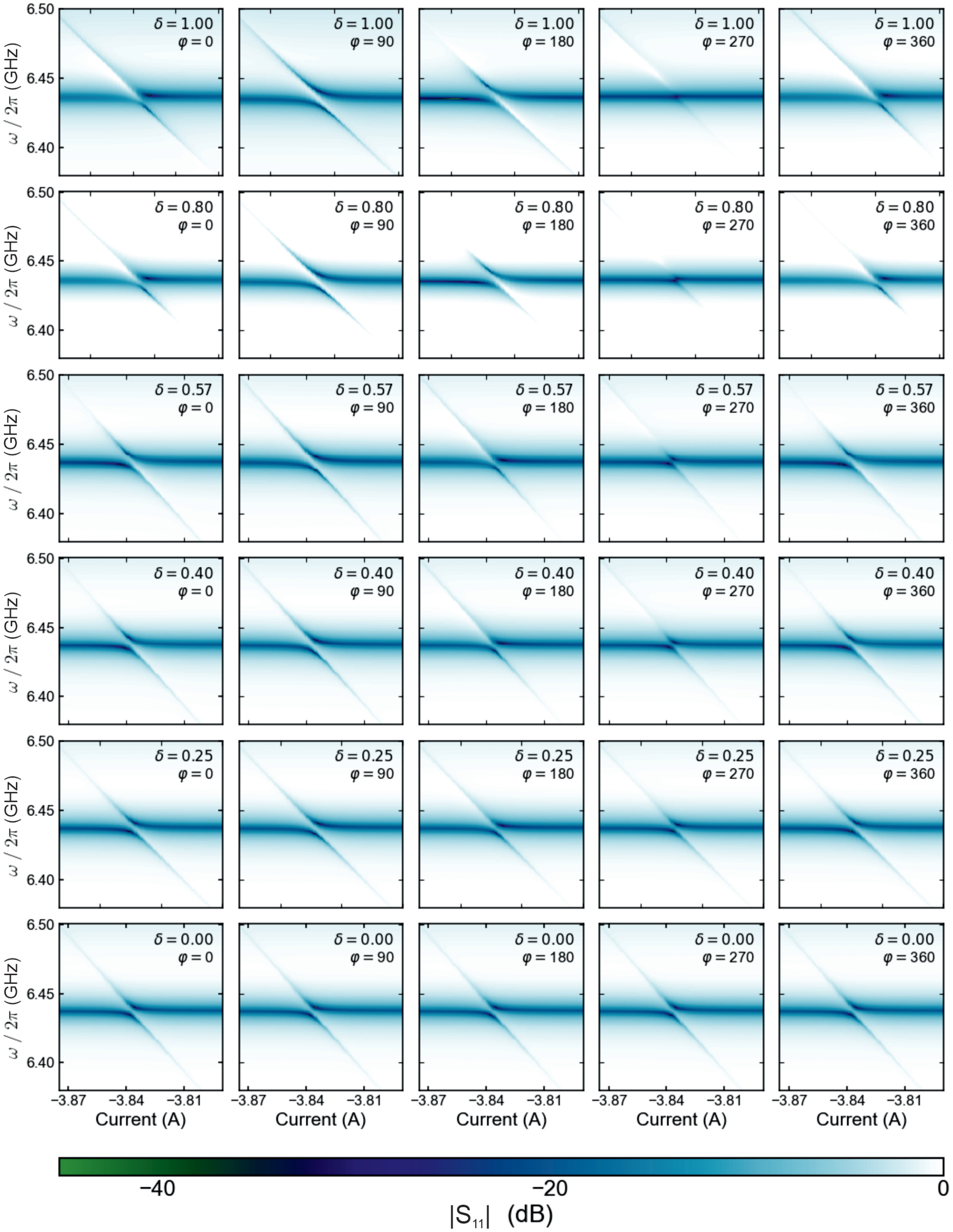

Fig S13. Experimental data using 0.25mm diameter YIG sphere for $\mathbf{H_0} = -\hat{\mathbf{z}}H_0$.

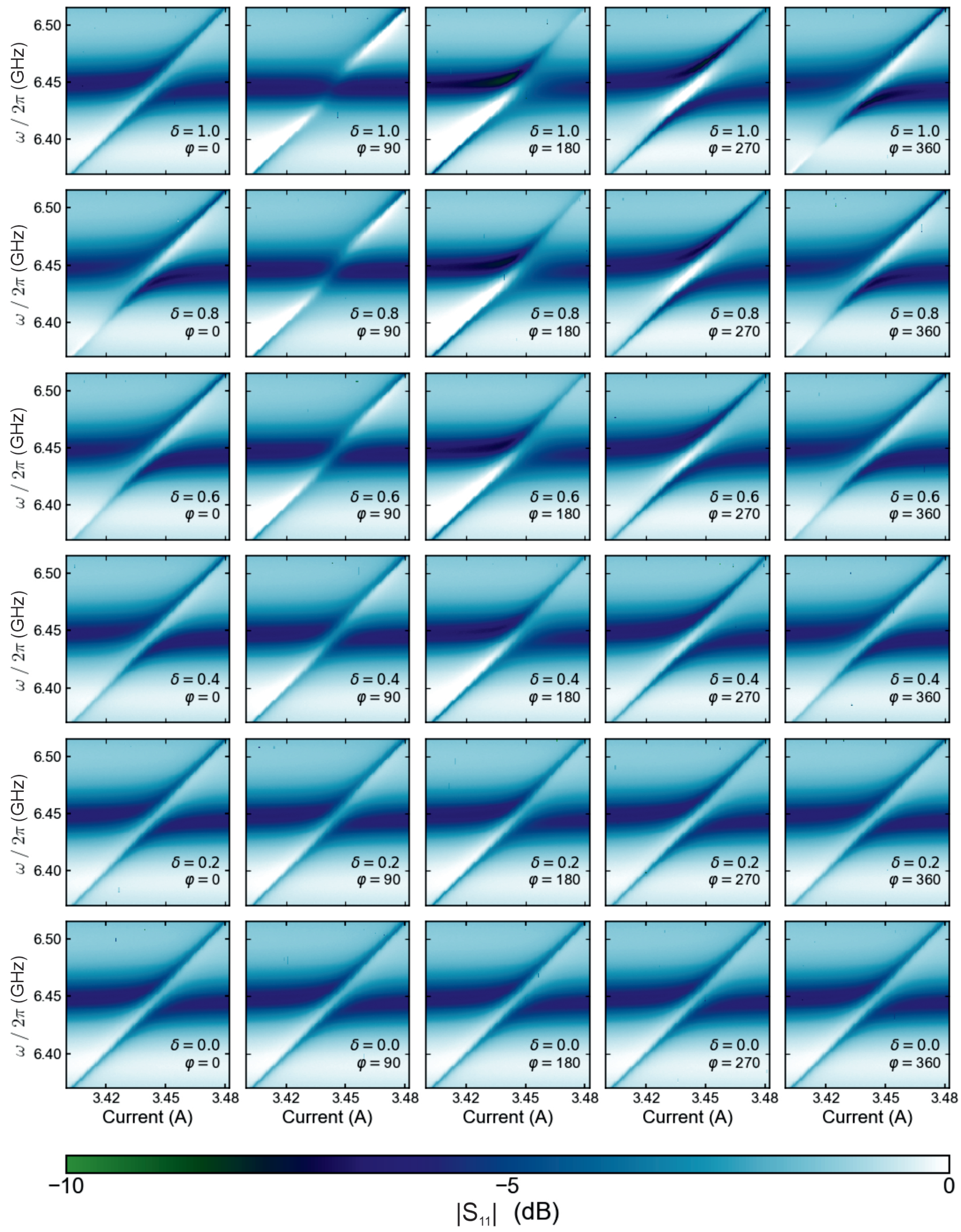

Fig S14. Experimental data using 0.5mm diameter YIG sphere for $\mathbf{H_0} = +\hat{\mathbf{z}}H_0$

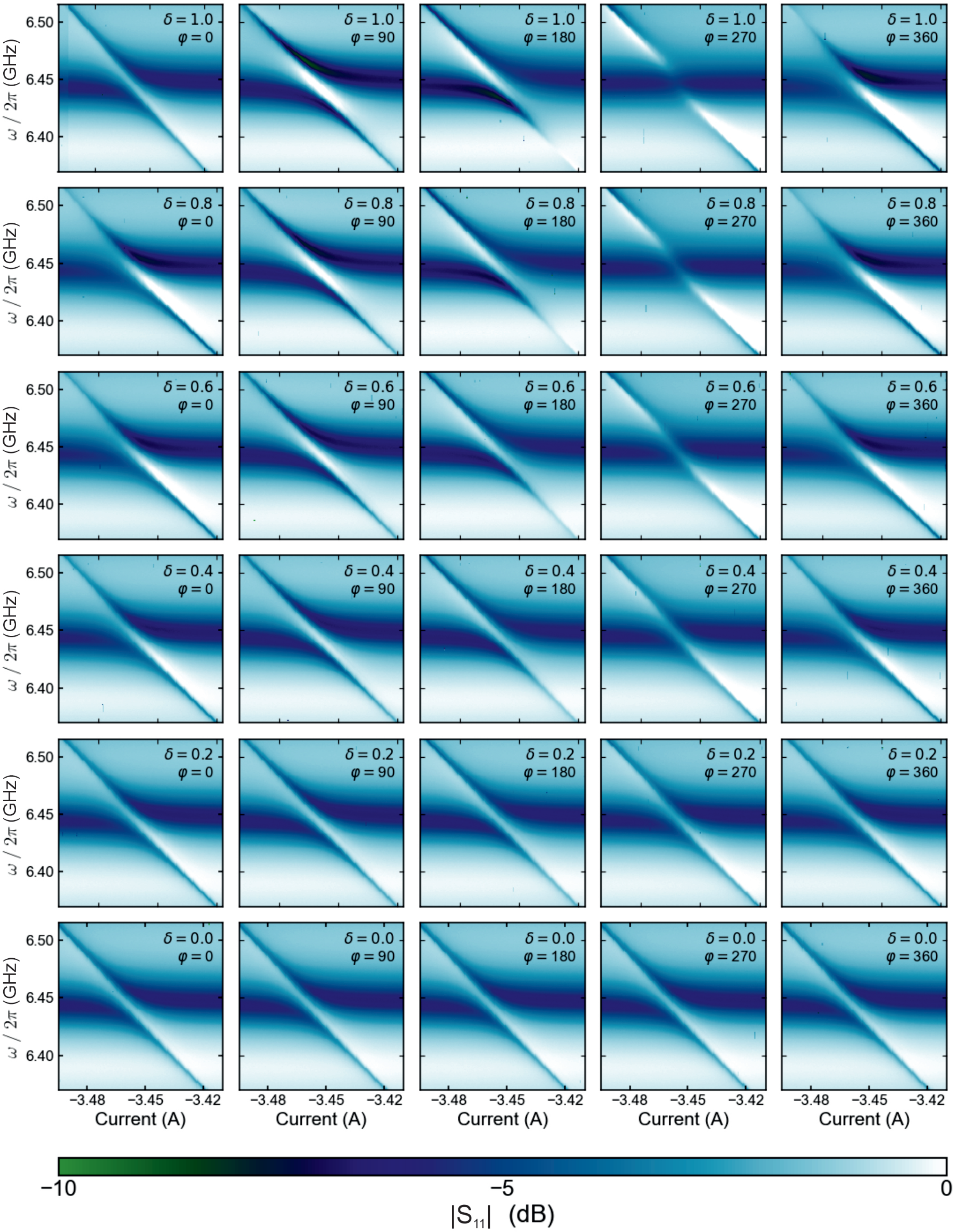

Fig S15. Experimental data using 0.5mm diameter YIG sphere for $\mathbf{H_0} = +\hat{\mathbf{z}}H_0$

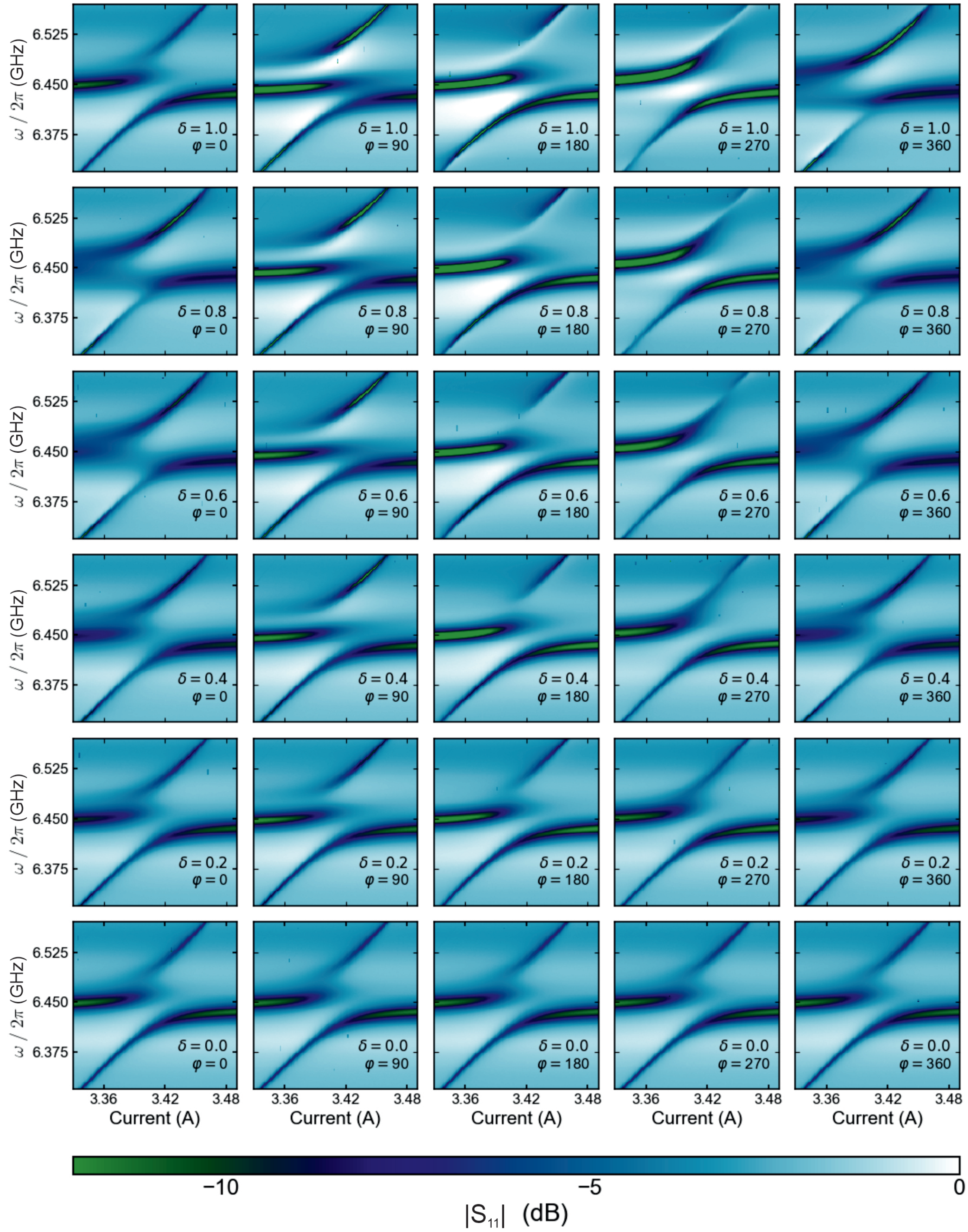

Fig S16. Experimental data using 1mm diameter YIG sphere for $\mathbf{H_0} = +\hat{\mathbf{z}}H_0$

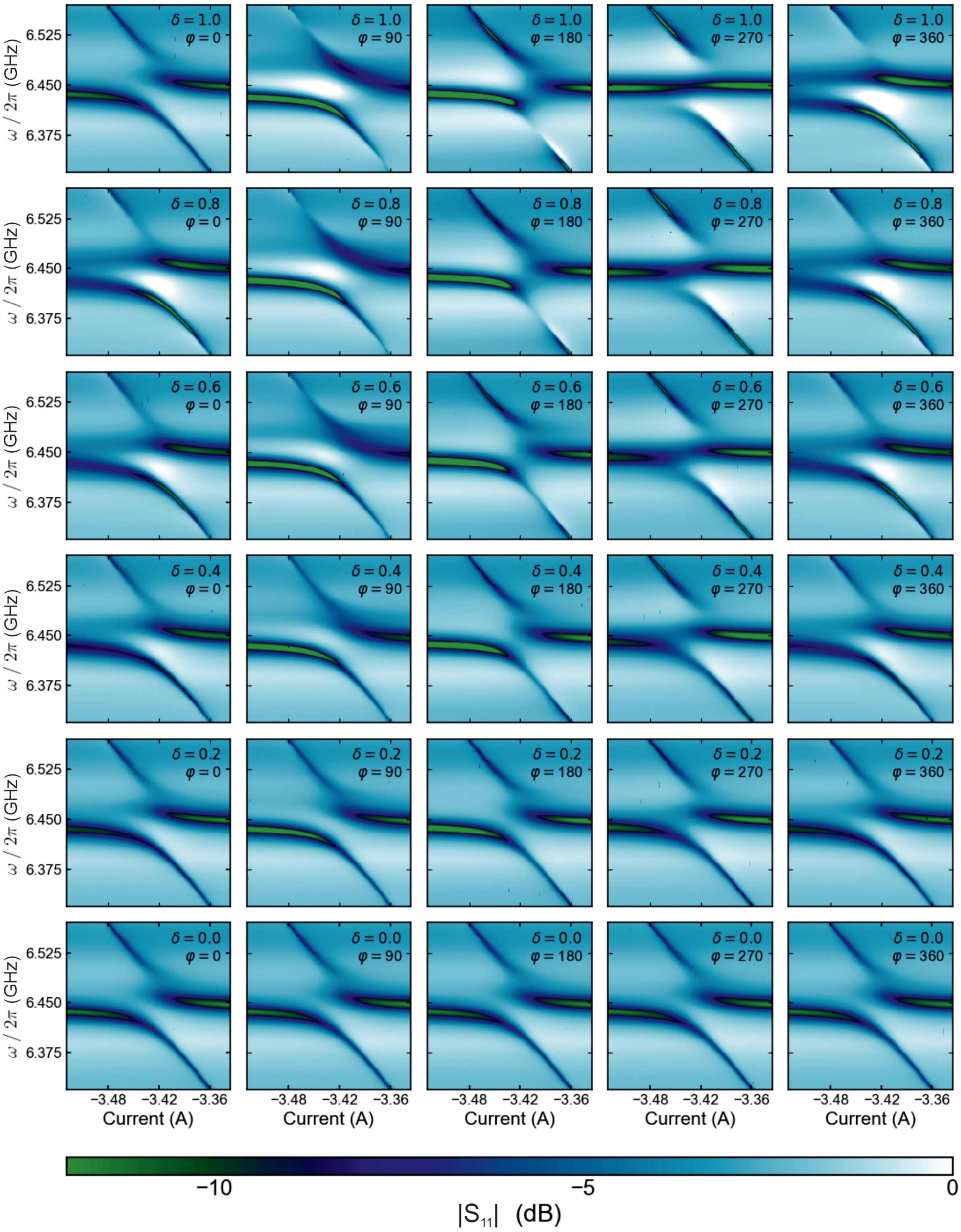

Fig S17. Experimental data using 1mm diameter YIG sphere for $\mathbf{H_0} = -\hat{\mathbf{z}}H_0$
